# Recent Updates on the Secondary Metabolites from *Fusarium* Fungi and Their Biological Activities (Covering 2019 to 2024)

**DOI:** 10.3390/jof10110778

**Published:** 2024-11-09

**Authors:** Prosper Amuzu, Xiaoqian Pan, Xuwen Hou, Jiahang Sun, Muhammad Abubakar Jakada, Eromosele Odigie, Dan Xu, Daowan Lai, Ligang Zhou

**Affiliations:** Department of Plant Pathology, College of Plant Protection, China Agricultural University, Beijing 100193, China; amuzuprosper07@cau.edu.cn (P.A.); xiaoqianpan@cau.edu.cn (X.P.); xwhou@cau.edu.cn (X.H.); jiahangsun@cau.edu.cn (J.S.); jakada@cau.edu.cn (M.A.J.); eromoseleodigie@cau.edu.cn (E.O.); cauxudan@cau.edu.cn (D.X.); dwlai@cau.edu.cn (D.L.)

**Keywords:** secondary metabolites, nitrogen-containing metabolites, polyketides, terpenoids, *Fusarium* fungi, biological activities, plant pathogenic fungi, plant endophytic fungi, marine-derived fungi, soil-derived fungi

## Abstract

*Fusarium* species are commonly found in soil, water, plants, and animals. A variety of secondary metabolites with multiple biological activities have been recently isolated from *Fusarium* species, making *Fusarium* fungi a treasure trove of bioactive compounds. This mini-review comprehensively highlights the newly isolated secondary metabolites produced by *Fusarium* species and their various biological activities reported from 2019 to October 2024. About 276 novel metabolites were revealed from at least 21 *Fusarium* species in this period. The main metabolites were nitrogen-containing compounds, polyketides, terpenoids, steroids, and phenolics. The *Fusarium* species mostly belonged to plant endophytic, plant pathogenic, soil-derived, and marine-derived fungi. The metabolites mainly displayed antibacterial, antifungal, phytotoxic, antimalarial, anti-inflammatory, and cytotoxic activities, suggesting their medicinal and agricultural applications. This mini-review aims to increase the diversity of *Fusarium* metabolites and their biological activities in order to accelerate their development and applications.

## 1. Introduction

*Fusarium* is a widely distributed group of filamentous ascomycetes belonging to Sordariomycetes, Hypocreales, and Nectriaceae [1]. Some *Fusarium* species are among the most important toxigenic plant pathogenic fungi to infect crops such as wheat, barley, oats, rice, maize, potato, asparagus, mango, banana, grasses, and other food and feed grains. The toxic metabolites (or called mycotoxins) produced by pathogenic *Fusarium* fungi have been considered the main pathogenic factors. The consumption of these mycotoxin-contaminated foods may induce acute and long-term chronic diseases in humans and animals [2,3,4].

In addition to phytopathogenic *Fusarium* fungi that produce mycotoxins, it has been revealed that *Fusarium* fungi can grow on a wide range of substrates and are commonly found in soil, water, plants, and animals either on the continent or in the ocean. They have a variety of secondary metabolites with multiple biological activities that make *Fusarium* fungi a treasure trove of bioactive compounds [5,6]. Therefore, studies on the secondary metabolites of *Fusarium* fungi and their biological activities have always received much attention [7,8,9,10,11,12,13].

Over the past decades, many significant advances, such as the identification of bioactive metabolites [14,15,16], mycotoxins and their detoxification [2,17,18], biosynthesis and regulation [19,20], and development and applications [21,22] of *Fusarium* secondary metabolites, have been achieved. *Fusarium* metabolites and their biological activities have been well-reviewed up to 2019 [11,14,15,16,23]. Since then, many newly isolated metabolites have been identified in *Fusarium* fungi. In this mini-review, we focus on the recently identified novel secondary metabolites, along with their biological activities reported from 2019 to October 2024, to increase the diversity of *Fusarium* metabolites and their biological activities as well as to speed up their development and applications.

## 2. Nitrogen-Containing Metabolites and Their Biological Activities

The nitrogen-containing metabolites from *Fusarium* fungi mainly include amines, amides, cyclic peptides, pyridines, pyridones, and indole and imidazole analogs. The nitrogen-containing metabolites and their biological activities along with *Fusarium* species and their origins are shown in Table 1.

### 2.1. Amines

**Figure 1:**
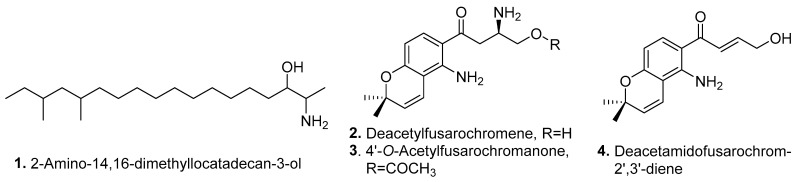
Structures of the amines (**1**–**4**) isolated from *Fusarium* fungi.

Four new amines (**1**–**4**) were isolated from *Fusarium* fungi, and their structures are shown in Figure 1.

2-Amino-14,16-dimethyloctadecan-3-ol (2-AOD-3-ol, **1**) was characterized as a new sphingolipid analog isolated from the plant pathogen *F. avenaceum*. 2-AOD-3-ol (**1**) showed moderate cytotoxic activity against HepG2 cells [24].

Two fusarochromenes, namely, deacetylfusarochromene (**2**) and 4′-*O*-acetylfusarochromanone (**3**), were isolated from the entomogenous fungus *Fusarium* sp. FKI-9521 derived from the feces of a stick insect *Ramulus mikado*. Both compounds showed moderate antimalarial activity against chloroquine-sensitive and -resistant *Plasmodium falciparum* strains, with the median inhibitory concentration (IC_50_) values ranging from 0.08 to 6.35 µM [25]. Deacetyl fusarochromene (**2**) was also isolated from the marine fungus *F. equiseti* UBOCC-A-117302 and showed obviously cytotoxic activities on RPE-1, HCT-116, and U2OS cells, with IC_50_ values of 0.176, 0.087, and 0.896 μM, respectively [26].

Another fusarochromanone, namely, deacetamidofusarochrom-2′,3′-diene (**4**), was isolated from the marine fungus *F. equiseti* UBOCC-A-117302, which was derived from a seawater sample collected in the coastal region of Chile. Deacetamidofusarochrom-2′,3-diene (**4**) showed antibacterial activity against *Listeria monocytogenes*, with a minimum inhibitory concentration (MIC) value of 125 μM, and also exhibited cytotoxic activities on RPE-1, HCT-116, and U2OS cells with IC_50_ values of 10.03, 13.73, and 13.18 μM, respectively [26].

### 2.2. Amides

**Figure 2:**
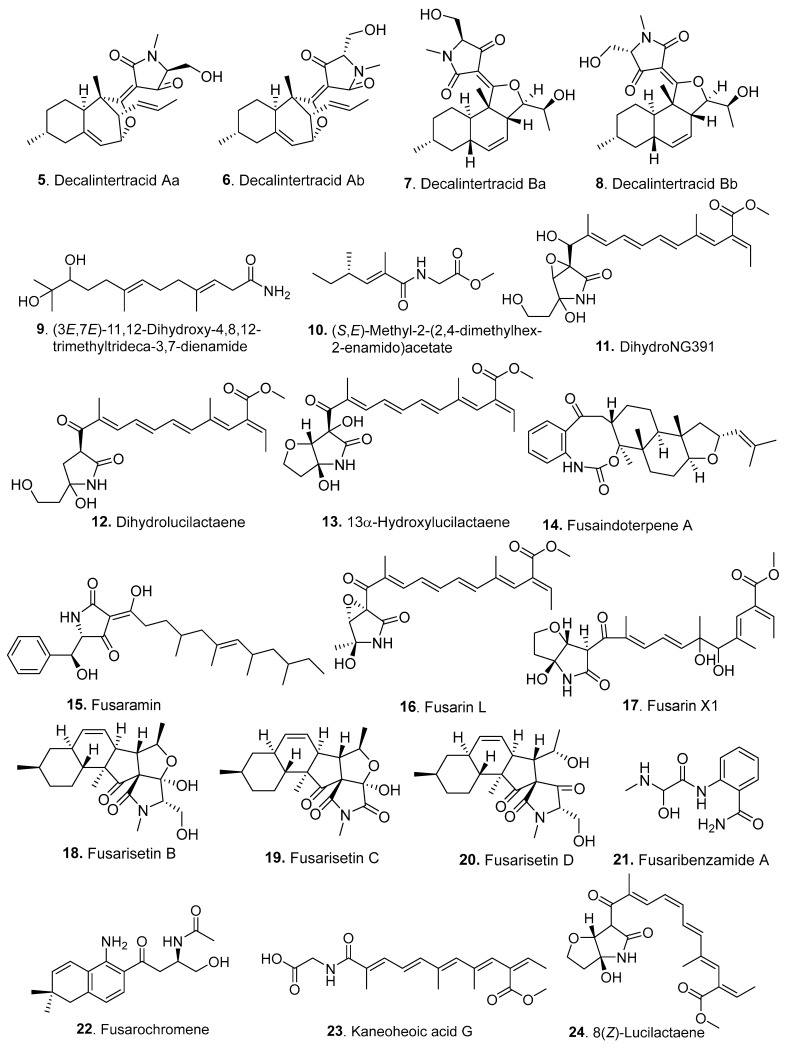

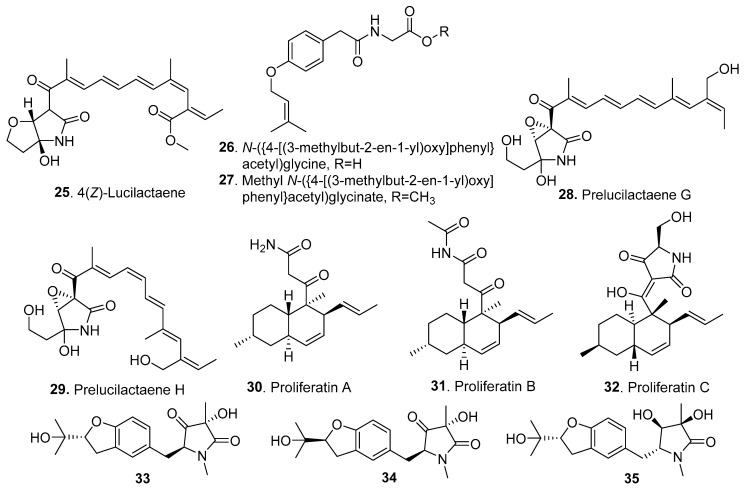
Structures of the amides (**5**–**35**) isolated from *Fusarium* fungi.

Thirty-one new amides (**5**–**35**) were isolated from *Fusarium* fungi, with their structures shown in Figure 2.

Decalintertracids A (**5**/**6**) and B (**7**/**8**) were two pairs of 3-decalinoyltetramic acid *E*/*Z* diastereomers (3DTAs), namely, decalintertracids Aa (**5**), Ab (**6**), Ba (**7**), and Bb (**8**). They were isolated from the endophytic fungus *F. equiseti* D39 derived from *Suaeda salsa* (Chenopodiaceae) collected in Qingdao, China. Decalintetracid A (**5**/**6**) was isolated as an inseparable mixture of two isomers with a ratio of 5:3. Although HPLC analysis of decalintetracid A (**5**/**6**) through both ODS and chiral column showed that it afforded a baseline separation of the two isomers, the attempts to separate them failed due to spontaneous isomerization between the two compounds. The two isomers have the same molecular formula of C_22_H_29_NO_4_. Similarly, decalintetracid B (**7**/**8**) was also obtained as a pair of inseparable isomers with a ratio of 5:3 and was determined to have the same molecular formula C_22_H_32_NO_5_ based on the HRESIMS data. Both compounds exhibited phytotoxic activity toward *Amaranthus retroflexus* and *A. hybrid*, indicating their potential as natural herbicides [27].

One sesquiterpene-derived amide, namely, (3*E*,7*E*)-11,12-dihydroxy-4,8,12-trimethyltrideca-3,7-dienamide (**9**), was isolated from plant endophytic fungus *Fusarium* sp. HJT-P-2, which was isolated from *Rhodiola angusta* (Crassulaceae) on Changbai Mountain, Jilin Province, China [28].

(*S*,*E*)-Methyl-2-(2,4-dimethylhex-2-enamido)acetate (**10**) is an amide isolated from the co-culture of two endophytic fungal species, *F. oxysporum* R1 and *Aspergillus fumigatus* D, derived from two traditional medicinal plants, *Edgeworthia chrysantha* Lindl. (Thymelaeaceae) and *Rumex madaio* Makino (Polygonaceae), respectively [29].

Three amides (**11**–**13**) were isolated from the cultures of *Fusarium* sp. RK97-94 treated with the metabolism regulator NPD938. The endophytic fungus *Fusarium* sp. RK97-94 was previously isolated from the leaves of an unidentified plant collected at Mt. Inasa, Nagasaki Prefecture, Japan. Three amides were identified as dihydroNG391 (**11**), dihydrolucilactaene (**12**), and 13α-hydroxylucilactaene (**13**) and showed antimalarial activities against *Plasmodium falciparum*, with IC_50_ values of 62 μM, 0.0015 μM, and 0.68 μM, respectively. The structure–activity relationship (SAR) showed that the epoxide was highly detrimental to antimalarial activity [30].

Fusaindoterpene A (**14**) was isolated from the marine-derived fungus *Fusarium* sp. L1, which was isolated from the inner tissue of the sea star *Acanthaster planci* collected from the Xisha Islands in China [31].

Fusaramin (**15**) was isolated from the soil-derived fungus *Fusarium* sp. FKI-7550. Fusaramin (**15**) showed antibacterial activity against some Gram-positive and Gram-negative bacteria and decreased the growth of *Saccharomyces cerevisiae* through the inhibition of ATP synthesis via oxidative phosphorylation in mitochondria [32].

Fusarin L (**16**) was isolated from the marine-derived fungus *F. solani* 7227, which was isolated from a seawater sample collected in the South China Sea [33].

Fusarin X1 (**17**) was a hybrid of polyketide with non-ribosomal peptide, which was isolated from the *Fusarium* head blight pathogen *F. graminearum*. Fusarin X1 (**17**) showed moderate cytotoxic activity against three tumor cell lines [34].

Fusarisetin B (**18**) was isolated from the entomogenous fungus *F. equiseti* LGWB-9, isolated from *Harmonia axyridis* (Coccinellidae). Fusarisetin B (**18**) showed cytotoxicity against MCF-7, MGC-803, HeLa, and Huh-7 cell lines, with the IC_50_ values ranging from 2.4 to 69.7 μg/mL. Cell invasion, migration, DAPI staining, and flow cytometry experiments were carried out to examine the effects of fusarisetin B (**18**) on MGC-803 cells. Western blot results showed that fusarisetin B (**18**) could induce MGC-803 apoptosis through the up-regulation of Bax and down-regulation of Bcl-2 [35].

Fusarisetins C (**19**) and D (**20**) were isolated from the marine-derived fungus *F. equiseti* D39, which was from a piece of fresh tissue obtained from the inner part of an unidentified plant collected from the intertidal zone of the Yellow Sea, Qingdao, China. Fusarisein D (**20**) was identified as the first fusarisetin to possess an unprecedented carbon skeleton with a tetracyclic ring system comprising a decalin moiety (6/6) and a tetramic acid moiety [36].

An aminobenzamide derivative, namely, fusaribenzamide A (**21**), was isolated from the endophytic fungus *Fusarium* sp. isolated from the roots of *Mentha longifolia* (Labiatae). Fusaribenzamide A (**21**) possessed significant antifungal activity toward *Candida albicans*, with a higher minimum inhibitory concentration (MIC) value of 11.9 μg/disc compared to the positive control nystatin (MIC, 4.9 μg/disc) [37].

Fusarochromene (**22**) was isolated from plant pathogenic fungus *F. sacchari*. It was considered a tryptophan-derived metabolite [38].

Kaneoheoic acid G (**23**) was isolated from the marine-derived fungus *F. graminearum* FM1010, which was isolated from shallow-water volcanic rock known as “live rock” at Richardson’s Beach, Hilo, Hawaii. Biogenetically, kaneoheoic acid G (**23**) could be catalyzed by NRPS-PKS. Kaneoheoic acid G (**23**) showed strong antibacterial activity against *Staphylococcus aureus* and methicillin-resistant *S. aureus* [39].

Two photosensitive geometrical isomers 8(*Z*)-lucilactaene (**24**) and 4(*Z*)-lucilactaene (**25**) of lucilactaene were isolated from *Fusarium* sp. QF001, a plant endophytic fungus from the roots of *Scutellariae baicalensis* (Labiatae). Both compounds showed anti-inflammatory activity by inhibiting NO production and suppressing pro-inflammatory cytokine expression in LPS-stimulated macrophage cells [40].

Two prenylated glycine derivatives, namely, *N*-({4-[(3-methylbut-2-en-1-yl)oxy]phenyl}acetyl)glycine (**26**) and methyl *N*-({4-[(3-methylbut-2-en-1-yl)oxy]phenyl}acetyl)glycinate (**27**), were isolated from the marine-derived fungus *Fusarium* sp. TW56-10, which was derived from hydrothermal vent sediment collected in Kueishantao, Taiwan [41].

Prelucilactaenes G (**28**) and H (**29**) were isolated from *Fusarium* sp. RK97-94 and showed antimalarial activity [42].

Froliferatins A (**30**), B (**31**), and C (**32**) were isolated from the stroma-derived fungus *F. proliferatum* isolated from *Cordycep sinensis*. The three metabolites (**30**–**32**) all showed anti-inflammatory activity by suppressing lipopolysaccharide-induced inflammation via inhibition of the NF-κB and MAPK signaling pathways [43].

Three unnamed pyrrolidinone analogs **33**–**35** were isolated from the endophytic fungus *F. decemcellualre* F25, which was derived from the stems of the medicinal plant *Mahonia fortunei* (Berberidaceae) collected in Qindao, China [44].

### 2.3. Cyclic Peptides

**Figure 3:**
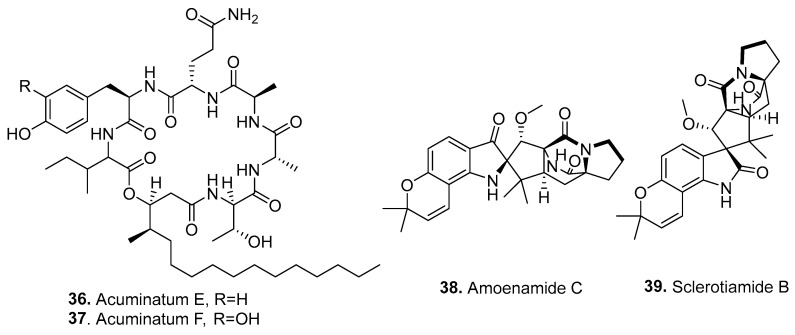

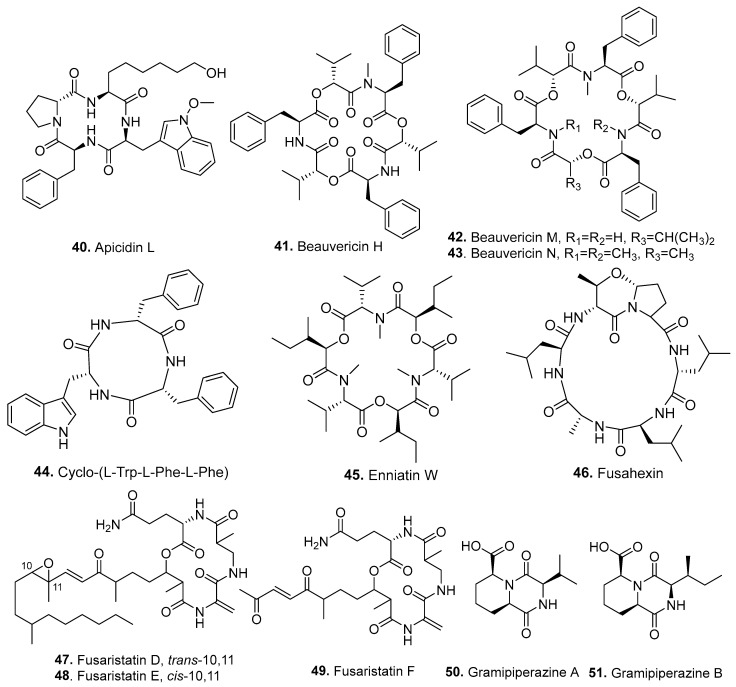
Structures of the cyclic peptides (**36**–**51**) isolated from *Fusarium* fungi.

Cyclic peptides are cyclic compounds formed mainly by the amide bonds between either proteinogenic or non-proteinogenic amino acids. Some fungal cyclic peptides are called cyclic depsipeptides, in which the corresponding lactone bonds replace amide groups due to the presence of a hydroxylated carboxylic acid in the peptide structure. Fifteen new amines (**36**–**51**) were isolated from *Fusarium* fungi, with their structures shown in Figure 3.

Two cyclic lipopeptides acuminatums, E (**36**) and F (**37**), were isolated from a corn culture of endophytic *F. lateritium* HU0053 derived from *Adenanthera pavonlna* (Leguminosae). Their structures were elucidated by spectroscopy and advanced Marfey’s amino acid analysis. All compounds exhibited antifungal activities against *Penicillium digitatum*. Acuminatum F (**37**), containing an unusual 3, 4-dihydroxy-phenylalanine unit, displayed the strongest antifungal activities, with an inhibition zone of 6.5 mm at a dose of 6.25 μg. Acuminatum F (**37**) may be a potential environmentally friendly preservative for citrus fruits [45].

Two cyclodipeptides containing an angularly prenylated indole moiety, namely, amoenamide C (**38**) and sclerotiamide B (**39**), were isolated from the endophytic fungus *F. sambucinum* TE-6L residing in *Nicotiana tabacum* (Solanaceae). Both compounds showed potent inhibitory effects on pathogenic bacteria and fungi. In addition, sclerotiamide B (**39**) exhibited remarkable larvicidal activity against first-instar larvae of the cotton bollworm (*Helicoverpa armigera*), with a mortality rate of 70.2% [46].

Apicidin L (**40**) was a cyclic tetrapeptide isolated from the rice pathogen *F. fujikuroi* IMI58289. The cytotoxic effects of apicidin L (**40**) were tested on three different cell lines. Cytotoxic effects were exhibited only at high concentrations in rat myoblast cells, with an IC_50_ value of 20 μM. In addition, apicidin L (**40**) exhibited in vitro antimalarial activity against *Plasmodium falciparum*, with an IC_50_ value of 2.1 μM [47].

Beauvericin H (**41**) is a cyclic hexadepsipeptide isolated from the ethanol extract of a solid culture of the plant endophytic fungus *Fusarium* sp. DCJ-A. Beauvericin H (**41**) and known isolated cyclic hexadepsipeptides exhibited cytotoxic activities against five human cancer cell lines, with IC_50_ values ranging from 1.379 to 13.12 μM [48].

Two cyclic hexadepsipeptides, namely, beauvericins M (**42**) and N (**43**), were isolated from the endophytic fungus *Fusarium* sp., which was derived from the stem of a tea plant (*Camelia sinensis*) [49].

One cyclo-tripeptide, namely, cyclo-(L-Trp-L-Phe-L-Phe) (**44**), was isolated from the plant endophytic fungus *F. proliferatum* T2-10 and showed cytotoxic and antibacterial activities [50].

One cyclic hexadepsipeptide, namely, enniatin W (**45**), was isolated from the endophytic fungus *F. oxysporum* LHS-P1-3 from the roots of *Arachis hypogaea* (Leguminosae). Enniatin W (**45**) exhibited cytotoxic activity against tumor cells of HepG2 and HeLa lines [51].

The overexpression of the *NRPS4* gene in the plant pathogenic fungus *F. graminearum* led to the discovery of a new cyclic hexapeptide, fusahexin (**46**), with the amino acid sequence cyclo-(D-Ala-L-Leu-D-*allo*-Thr-L-Pro-D-Leu-L-Leu) [52].

Three lipodepsipeptides, fusaristatins D (**47**), E (**48**), and F (**49**), were obtained from the solid rice cultures of *Fusarium* sp. BZCB-CA, an endophytic fungus derived from the Chinese medicinal plant *Bothriospermum chinense* (Boraginaceae) [53].

Two diketopiperazines, namely, gramipiperazines A (**50**) and B (**51**), were isolated from the marine-derived fungus *F. graminearum* FM1010, which was isolated from shallow-water volcanic rock known as “live rock” at Richardson’s Beach, Hilo, Hawaii. Biogenetically, gramipiperazine A (**50**) should be derived from 2-amino-5-hydroxyadipic acid and valine, and gramipiperazine B (**51**) should be derived from 2-amino-5-hydroxyadipic acid and isoleucine. Gramipiperazine B (**51**) showed weak antibacterial activity against *Staphylococcus aureus* and methicillin-resistant *S. aureus* [39].

### 2.4. Pyridines

**Figure 4:**
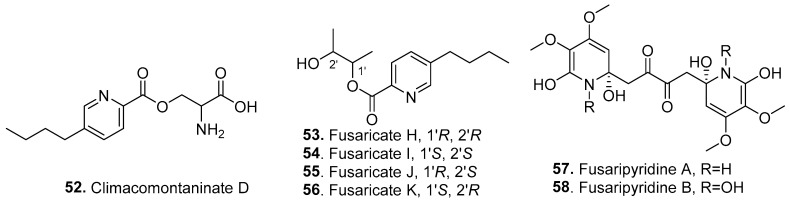
Structures of the pyridines (**52**–**58**) isolated from *Fusarium* fungi.

Seven new pyridine analogs (**52**–**58**) were isolated from *Fusarium* fungi, with their structures shown in Figure 4.

Climacomontaninate D (**52**) was isolated from the entomogenous fungus *Fusarium* sp. LGWB-7 from *Harmonia axyridis* [54].

Four fusaric acid derivatives, fusaricates H (**53**), I (**54**), J (**55**), and K (**56**), were isolated from the mangrove-derived endophytic fungus *F. solani* HDN15-410, which was isolated from the roots of *Rhizophora apiculata* Blume (Rhizophoraceae) [55].

Two dimeric pyridine derivatives, fasaripyridines A (**57**) and B (**58**), were identified from the marine-derived fungus *Fusarium* sp. LY019, which was obtained from the sponge *Suberea mollis* in the Red Sea. Both compounds possessed a previously unreported moiety, 1,4-bis(2-hydroxy-1,2-dihydropyridin-2-yl)butane-2,3-dione. They selectively inhibited the growth of *Candida albicans* with MIC values of 8.0 μM, while they were moderately active against *Staphylococcus aureus*, *Escherichia coli*, and HeLa cells [56].

### 2.5. Pyridones

**Figure 5:**
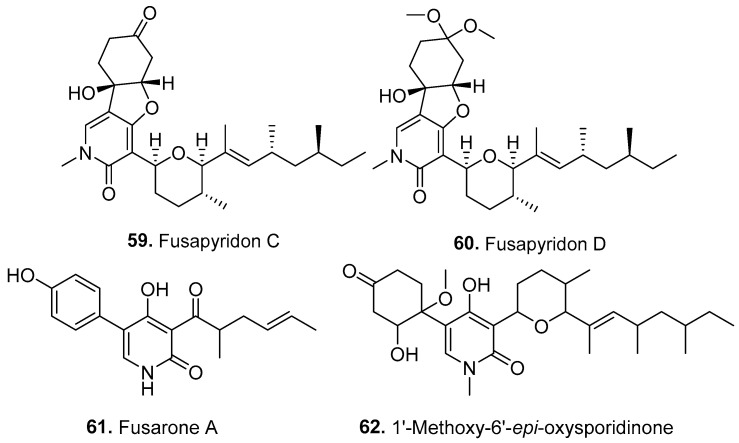
Structures of the pyridines (**59**–**62**) isolated from *Fusarium* fungi.

Four new pyridones (**59**–**62**) were isolated from *Fusarium* fungi, and their structures are shown in Figure 5.

Two tricyclic pyridone analogs, fusapyridons C (**59**) and D (**60**), were isolated from the entomopathogenic fungus *F. avenaceum* SYKC02-P-1. Both compounds showed inhibitory activity against human prostate cancer cells (PC-3 cell line) [57].

Fusarone A (**61**) was a 2-pyridone derivative isolated from the endophytic fungus *F. proliferatum* T2-10. Fusarone A (**61**) showed cytotoxic and antibacterial activities [50].

One pyridine derivative, 1′-methoxy-6′-*epi*-oxysporidinone (**62**), was isolated from the endophytic fungus *F. concentricum*, which was derived from the medicinal plant *Anoectochilus roxburghii* (Orchidaceae) [58].

### 2.6. Indole Analogs

**Figure 6:**
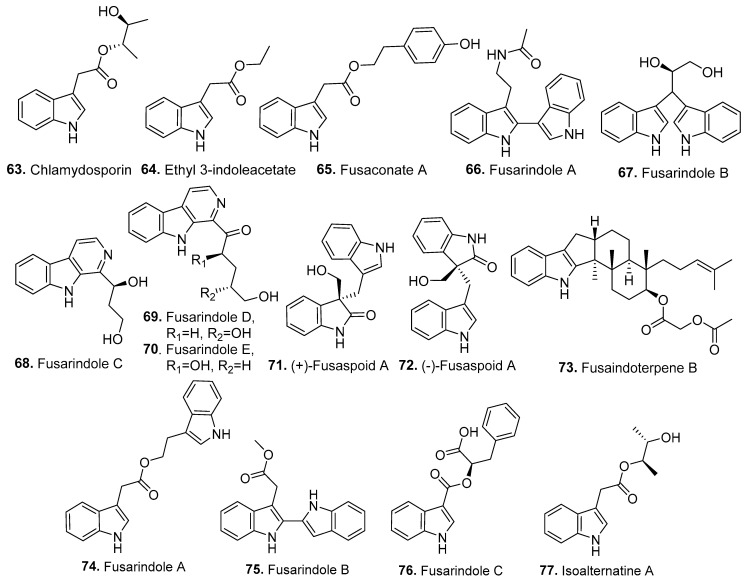
Structures of the indole analogs (**63**–**77**) isolated from *Fusarium* fungi.

Fifteen new indole analogs (**63**–**77**) were isolated from *Fusarium* fungi, and their structures are shown in Figure 6.

Chlamydosporin (**63**) was isolated from *F. chlamydosporum* derived from the roots of *Suaeda glauca* (Chenopodiaceae). Chlamydosporin (**63**) showed significant phytotoxic activity against the radicle growth of *Echinochloa crusgalli* (Gramineae) seedlings [59].

Ethyl 3-indoleacetate (**64**) is an indole derivative isolated from the endophytic fungus *F. proliferatum* T2-10. It showed cytotoxic and antibacterial activities [50].

Fusaconate A (**65**) is a tryptophan derivative isolated from the endophytic fungus *F. concentricum*, which was derived from the medicinal plant *Anoectochilus roxburghii* (Orchidaceae) [58].

Five indole analogs, namely, fusarindoles A (**66**), B (**67**), C (**68**), D (**69**), and E (**70**), were isolated from the marine-derived fungus *F. equiseti* LJ-1, which was derived from the soft coral *Sarcophyton tortuosum* collected in the South China Sea [60].

A pair of novel bisindole alkaloid enantiomers, namely, (+)-fusaspoid A (**71**) and (−)-fusaspoid A (**72**), was isolated from the marine-derived fungus *Fusarium* sp. XBB-9, which was isolated from Hainan Sanya National Coral Reef Reserve in China [61].

Fusaindoterpene B (**73**), fusariumindoles A–C (**74**–**76**), and isoalternatine A (**77**) were isolated from the marine-derived fungus *Fusarium* sp. L1, which was isolated from the inner tissue of a sea star *Acanthaster planci* collected from the Xisha Islands in China. Among the compounds, fusaindoterpene B (**73**) displayed the strongest inhibitory activity against the Zika virus (ZIKV) in a standard plaque assay, with an IC_50_ value of 7.5 μM [31].

### 2.7. Imidazole Analogs

**Figure 7:**
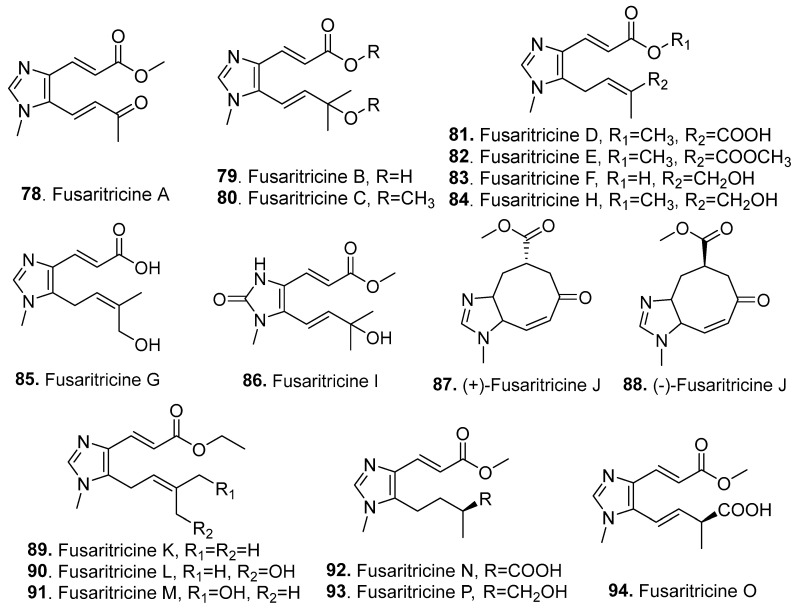
Structures of the imidazole analogs (**78**–**94**) isolated from *Fusarium* fungi.

Seventeen new imidazole analogs (**78**–**94**) were isolated from *Fusarium* fungi, and their structures are shown in Figure 7.

Nine imidazole derivatives, namely, fusaritricines A–I (**78**–**86**), were isolated from the plant endophytic fungus *F. tricinctum*, which was derived from kiwi (*Actinidia chinensis*) plants (Actinidiaceae). Among these imidazole analogs, fusaritricines B (**79**), C (**80**), and I (**86**) showed strong antibacterial activity against *Pseudomonas syringae* pv. *actinidiae* (Psa), with MIC values ranging from 25 to 50 μg/mL [62]. Another eight imidazole derivatives, namely, (+)-fusaritricine J (**87**), (−)-fusaritricine J (**88**), and fusaritricines K–P (**89**–**94**) were further isolated from *F. tricinctum.* Among these compounds, (+)-fusaritricine J (**87**), (−)-fusaritricine J (**88**), fusaritricines M (**91**) and N (**92**) showed comparably strong antibacterial activities against Psa, with MIC values of 25–50 μg/mL. Further cell membrane permeability results suggested that the most active fusaritricine M (**91**) could destroy the bacterial cell wall structure [63]. These imidazole derivatives were only found in the genus *Fusarium* and showed strong antibacterial activity, which indicated that the imidazole derivatives may have applications as potential antibacterial agents. The endophytic fungus *F. tricinctum* could also be considered a biocontrol agent for managing the kiwi fruit canker disease. In addition, the physiological, ecological, and chemotaxonomic significance of imidazole derivatives, as well as their biosynthesis, should be studied in detail.

### 2.8. Other Nitrogen-Containing Metabolites

**Figure 8:**
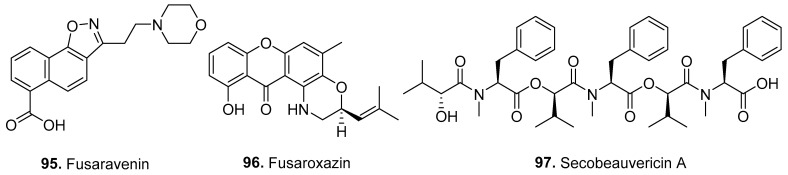
Structures of the other nitrogen-containing metabolites (**95**–**97**) isolated from *Fusarium* fungi.

The structures of other new nitrogen-containing metabolites isolated from *Fusarium* fungi are shown in Figure 8.

Fusaravenin (**95**) is a naphthoisoxazole formic acid connected to a morpholino carbon skeleton isolated from the soil-derived fungus *F. avenaceum* SF-1502 [64].

Fusarioxazin (**96**) is a 1,4-oxazine-xanthone derivative isolated from *F. oxysporum*, which is associated with the roots of *Vicia faba* (Leguminosae). Fusarioxazin (**96**) possessed significant antibacterial activity toward *Staphylococcus aureus* and *Bacillus cereus*, with MIC values of 5.3 mg/mL and 3.7 mg/mL, respectively. Furthermore, fusarioxazin (**96**) displayed a promising cytotoxic effect on HCT-116, MCF-7, and A549 cells, with IC_50_ values of 2.1 mM, 1.8 mM, and 3.2 mM, respectively [65].

Secobeauvericin A (**97**) is a linear hexadepsipeptide isolated from the endophytic fungus *Fusarium* sp., which was derived from the stem of a tea plant [49].

## 3. Polyketides and Their Biological Activities

The polyketides from *Fusarium* fungi mainly include pyrones, furanones, and quinones. The metabolites and their biological activities, along with *Fusarium* species and their origins, are shown in Table 2.

### 3.1. Pyrones

Pyrones are also called pyranones. Two types of pyrones named α- and γ-pyrones were derived from *Fusarium* fungi.

#### 3.1.1. α-Pyrones

**Figure 9:**
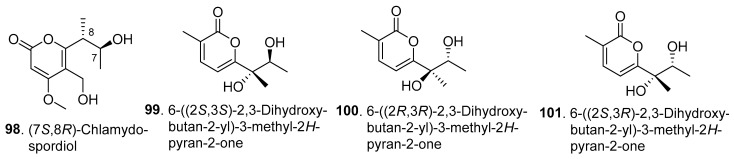

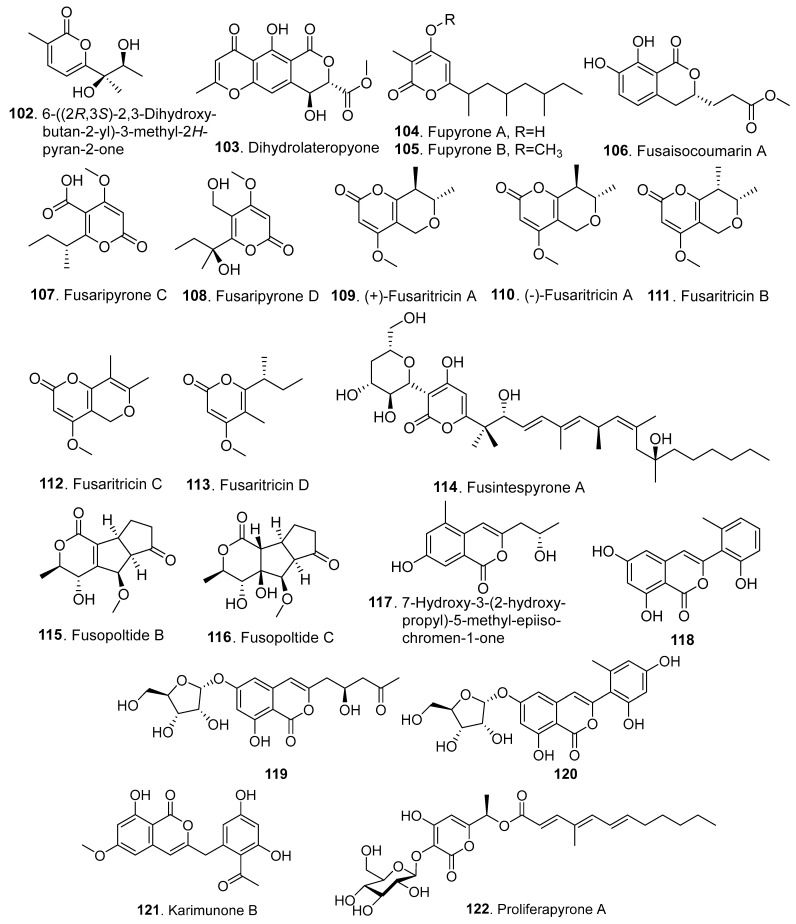
Structures of the α-pyrones (**98**–**122**) isolated from *Fusarium* fungi.

Twenty-five new α-pyrones (**98**–**122**) were isolated from *Fusarium* fungi, with their structures shown in Figure 9.

One new α-pyrone derivative, (7*S*,8*R*)-chlamydospordiol (**98**), was obtained from the endophytic fungus *Fusarium* sp. BZCB-CA, which was isolated from the Chinese medicinal plant *Bothriospermum chinense* (Boraginaceae) [53].

Four previously undescribed 2-pyrones sharing the same planar structure of 6-(2,3-dihydroxybutan-2-yl)-3-methyl-2*H*-pyran-2-one were isolated as two pairs of racemes by preparative HPLC and further as four epimers by subsequent chiral separation from the brown rice solid medium of *F. tricinctum*, an endophytic fungus of *Ligusticum chuanxiong*. They were identified as 6-((2*S*,3*S*)-2,3-dihydroxybutan-2-yl)-3-methyl-2*H*-pyran-2-one (**99**), 6-((2*R*,3*R*)-2,3-dihydroxybutan-2-yl)-3-methyl-2*H*-pyran-2-one (**100**), 6-((2*S*,3*R*)-2,3-dihydroxybutan-2-yl)-3-methyl-2*H*-pyran-2-one (**101**), and 6-((2*R*,3*S*)-2,3-dihydroxybutan-2-yl)-3-methyl-2*H*-pyran-2-one (**102**). The four 2-pyrones showed the same growth inhibition against HTC116, A549, and MV 4-11 cell lines, with IC_50_ values of 0.013, 0.014, and 0.039 μM, respectively [66].

Dihydrolateropyrone (**103**) is a lateropyrone derivative isolated from *F. tricinctum*; the endophytic fungus was derived from healthy, fresh rhizomes of *Aristolochia paucinervis* (Aristolochiaceae) [67].

Fupyrones A (**104**) and B (**105**) were isolated from the endophytic fungus *Fusarium* sp. F20, which was isolated from the stems of the medicinal plant *Mahonia fortunei* (Berberidaceae) [68].

Fusaisocoumarin A (**106**) was isolated from the plant endophytic fungus *F. verticillioides*, which was derived from the leaves of *Mentha piperita* (Labiatae). Fusaisocoumarin A (**106**) showed antifungal activity against *Aspergillus austroafricanus*, *A. versicolor*, *A. tubingensis*, *Phoma fungicola*, and *Candida albicans*, with MIC values ranging from 0.3 to 1.3 mg/mL [69].

Two α-pyrones, namely, fusaripyrones C (**107**) and D (**108**), were isolated from the endophytic fungus *Fusarium* sp. L33 derived from the leaves of *Dioscorea opposite* (Dioscoreaceae). Fusaripyrones C (**107**) and D (**108**) showed no obvious cytotoxic activity against five human tumor cell lines, including HL-60, A549, SMMC-7721, MDA-MB-231, and SW-480, at 40 μM, with an inhibition rate of less than 50% [70].

(+)-Fusaritricin A (**109**), (−)-fusaritricin A (**110**), and fusaritricins B (**111**), C (**112**), and D (**113**) were isolated from the plant endophytic fungus *F. tricinctum*, which was derived from kiwi (*Actinidia chinensis*) plants (Actinidiaceae). Fusaritricins A (**109**/**110**), B (**111**), and C (**112**) exhibited antibacterial activity against the plant pathogen *Pseudomonas syringae* pv. *actinidiae* (Psa), with MIC values of 128, 128, and 64 μg/mL, respectively [71].

Fusintespyrone A (**114**) was isolated from the intestinal fungus *Fusarium* sp. LE06 of the murine cecum. Fusintespyrone A (**114**) showed significant growth inhibition against *Aspergillus fumigatus*, *F. oxysporum*, and *Verticillium dahlia*, with MIC values in the range of 1.56–6.25 μg/mL [72].

Two α-pyrone analogs, fusopoltides B (**115**) and C (**116**), were isolated from *F. solani* B-18, which was isolated from the inner tissue of unidentified forest litter collected in the Mount Merapi Area, Indonesia [73].

7-Hydroxy-3-(2-hydroxy-propyl)-5-methyl-epiisochromen-1-one (**117**) was isolated from the marine-derived fungus *F. graminearum* FM1010, which was isolated from shallow-water volcanic rock known as “live rock” at Richardson’s Beach, Hilo, Hawaii [39].

Three unnamed isocoumarin analogs, **118**–**120**, were isolated from the endophytic fungus *F. decemcellualre* F25, which was derived from the stems of the medicinal plant *Mahonia fortune* (Berberidaceae) collected in Qindao, China [44].

One benzopyranone, namely, karimunone B (**121**), was isolated from the sponge-associated fungus *Fusarium* sp. KJMT.FP.4.3. Karimunone B (**121**) showed antibacterial activity against multidrug-resistant *Salmonella enterica* ser. Typhi with an MIC value of 125 μg/mL [74].

Proliferapyrone A (**122**) is an α-pyrone-polyketide glycoside isolated from the fermentation cultures of the plant pathogenic fungus *F. proliferatum*. Proliferapyrone A (**122**) did not exhibit phytotoxic activity and was considered the precursor of phytotoxins [75].

#### 3.1.2. γ-Pyrones

**Figure 10:**
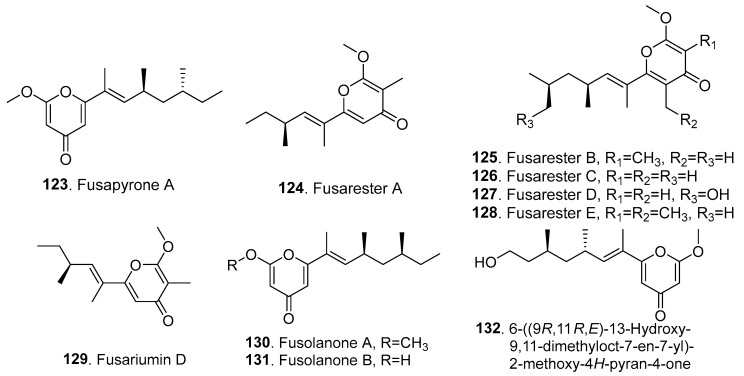
Structures of the γ-pyrones (**123**–**132**) isolated from *Fusarium* fungi.

Ten new γ-pyrones (**123**–**133**) were isolated from *Fusarium* fungi, and their structures are shown in Figure 10.

One γ-pyrone derivative, namely, fusapyrone A (**123**), was isolated from the rice fermentation cultures of *Fusarium* sp. CPCC 401218, a fungus collected from the desert. Fusapyone A (**123**) showed weak antiproliferative activity against HeLa cells with an IC_50_ value of 50.6 μM [76].

Five γ-pyrone-containing polyketides, namely, fusaresters A (**124**), B (**125**), C (**126**), D (**127**), and E (**128**), were isolated from the marine-derived fungus *Fusarium* sp. Hungcl, which was isolated from soil collected from the Futian Mangrove Reserve in Shenzhen, China. Among them, fusarester B (**125**) showed a weak inhibition rate of 56% against protein tyrosine phosphatase 1B (PTP1B) at 40 μM [77].

Fusariumin D (**129**) is a γ-pyrone-containing derivative isolated from *F. oxysporum* ZZP-R1, which was derived from the coastal plant *Rumex madio* Makino (Polygonaceae). Fusariumin D (**129**) was previously identified as a sesquiterpene ester [78], and later, the structure was revised as a γ-pyrone derivative [77]. This metabolite had moderate antibacterial activity against *Staphylococcus aureus*, with an MIC value of 25.0 μM [78].

Two γ-pyrone derivatives, fusolanones A (**130**) and B (**131**), were isolated from the mangrove-derived endophytic fungus *F. solani* HDN15-410, which was isolated from the roots of *Rhizophora apiculata* Blume (Rhizophoraceae). Fusolanone A (**130**) showed antibacterial activity against *Pseudomonas aeruginosa*, with MIC value of 26.4 μg/mL. Fusolanone B (**131**) showed strong antibacterial activity against *Monilia albican*, *Pesudomonas aeruginosa*, *Bacillus subtilis*, and *Vibrio parahaemolyticus*, with MIC values of 12.5 μg/mL, 12.5 μg/mL, 50 μg/mL, and 6.25 μg/mL, respectively [55].

One γ–pyrone, namely, 6-((9*R*,11*R*,*E*)-13-Hydroxy-9,11-dimethyloct-7-en-7-yl)-2-methoxy-4*H*-pyran-4-one (**132**), was isolated from the plant endophytic fungus *F. solani* JS-0169, which was obtained from the leaves of *Morus alba* (Moraceae). This γ–pyrone showed moderate neuroprotective activity [79].

### 3.2. Furanones

**Figure 11:**
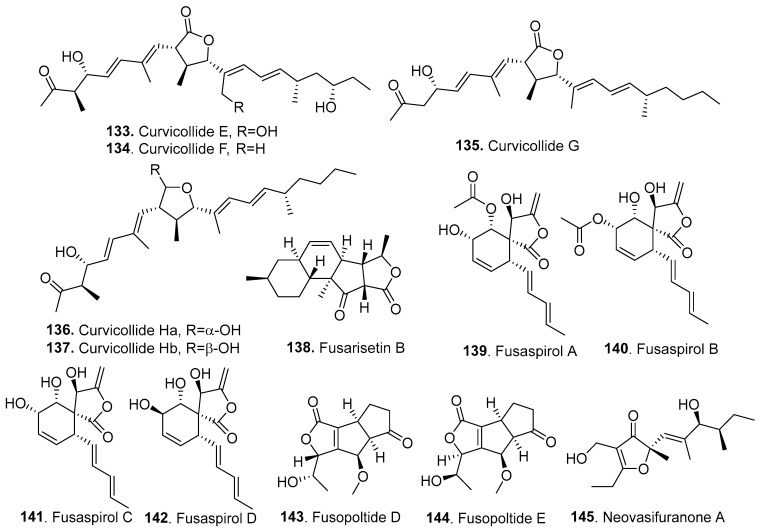

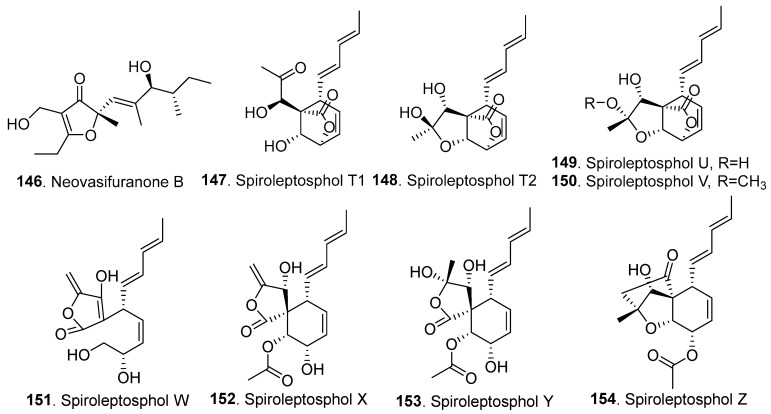
Structures of the furanones (**133**–**154**) isolated from *Fusarium* fungi.

Twenty-two new furanones (**133**–**154**) were isolated from *Fusarium* fungi, and their structures are shown in Figure 11.

Curvicollides E (**133**), F (**134**), G (**135**), Ha (**136**), and Hb (**137**) were isolated from the endophytic fungus *F. armeniacum*, which was derived from *Digitaia ciliaris* (Gramineae). Among the compounds, curvicollide E (**133**) showed the strongest cytotoxic activity against HeLa cells, with an IC_50_ value of 1.86 μg/mL [80].

Fusarisetin B (**138**) was isolated from the entomogenous fungus *F. equiseti* LGWB-9 from *Harmonia axyridis* and showed cytotoxicity against MCF-7, MGC-803, HeLa, and Huh-7 cell lines, with IC_50_ values ranging from 2.4 to 69.7 μg/mL [35].

Five polyketides with a γ-methylidene-spirobutanolide core, fusaspirols A (**139**), B (**140**), C (**141**), and D (**142**), were isolated from the plant endophytic fungus *F. solani* B-18, which was isolated from the inner tissue of unidentified forest litter collected in the area of Mount Merapi in Indonesia. Fusaspirol A (**139**) activated a signaling pathway in the osteoclastic differentiation of murine macrophage-derived RAW264.7 cells [81].

Two furanone analogs, fusopoltides D (**143**) and E (**144**), were isolated from *F. solani* B-18, which was isolated from the inner tissue of unidentified forest litter collected in the area of Mount Merapi in Indonesia [81].

Two diastereomeric polyketides, neovasifuranones A (**145**) and B (**146**), were obtained from solid rice cultures of the endophytic fungus *F. oxysporum* R1 associated with the Chinese medicinal plant *Rumex madaio* (Polygonaceae). Their absolute configurations were determined by combining modified Mosher’s reactions and chiroptical methods using time-dependent density functional theory electronic circular dichroism (TDDFT-ECD) and optical rotatory dispersion (ORD). Both compounds exhibited weak inhibitory antibacterial activity against *Helicobacter pylori* [82].

Eight furanones were isolated from the endophytic fungus *F. avenaceum* 05,001, which was isolated from Finnish grains. They were identified as spiroleptosphols T1 (**147**), T2 (**148**), U (**149**), V (**150**), W (**151**), X (**152**), Y (**153**), and Z (**154**) [83].

### 3.3. Quinones

**Figure 12:**
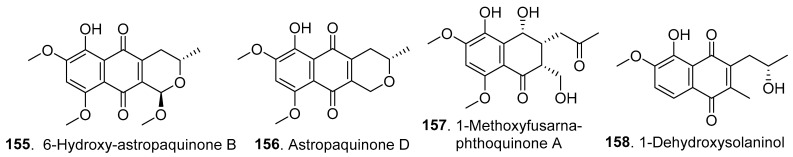

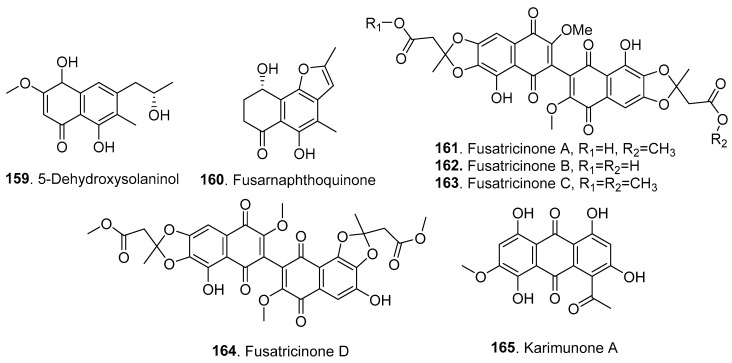
Structures of the quinones (**155**–**165**) isolated from *Fusarium* fungi.

Eleven new quinones (**155**–**165**) were isolated from *Fusarium* fungi, and their structures are shown in Figure 12.

Two naphthoquinone derivatives, 6-hydroxy-astropaquinone B (**155**) and astropaquinone D (**156**), were isolated from *F. napiforme*, an endophytic fungus isolated from the mangrove plant *Rhizophora mucronata* (Rhizophoraceae). Both 6-hydroxy-astropaquinone B (**155**) and astropaquinone D (**156**) exhibited moderate antibacterial activity against *Staphylococcus aureus* and *Pseudomonas aeruginosa* and phytotoxic activity against lettuce seedlings at a concentration of 30 μg/mL [84].

Four quinones, namely, 1-methoxylfusarnaphthoquinone A (**157**), 1-dehydroxysolaninol (**158**), 5-dehydroxysolaninol (**159**), and fusarnaphthoquinone D (**160**), were isolated from the endophytic fungus *Fusarium* sp. HJT-P-5 derived from *Rhodiola angusta* Nakai (Crassulaceae), which was obtained from Changbai Mountain in Jilin, China. Among them, 1-dehydroxysolaninol (**158**) showed the strongest cytotoxic activity against human colorectal cancer HCT116 cells, with an inhibitory rate of 64.39% at 100 μM [85].

*F. tricinctum*, an endophytic fungus that lives inside healthy, fresh rhizomes of *Aristolochia paucinervis* (Aristolochiaceae), created four new compounds called naphthoquinone dimers, namely, fusatricinones A–D (**161**–**164**). The antibacterial activity of these compounds was evaluated against the human pathogenic bacterial strains *Staphylococcus aureus* and *Pseudomonas aeruginosa*, but no activities were observed [67].

One new naphthoquinone, karimunone A (**165**), was isolated from the sponge-associated fungus *Fusarium* sp. KJMT.FP.4.3 [74].

### 3.4. Other Polyketides

**Figure 13:**
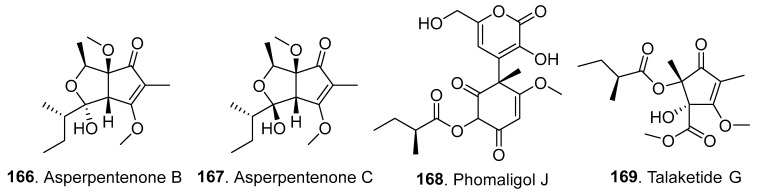

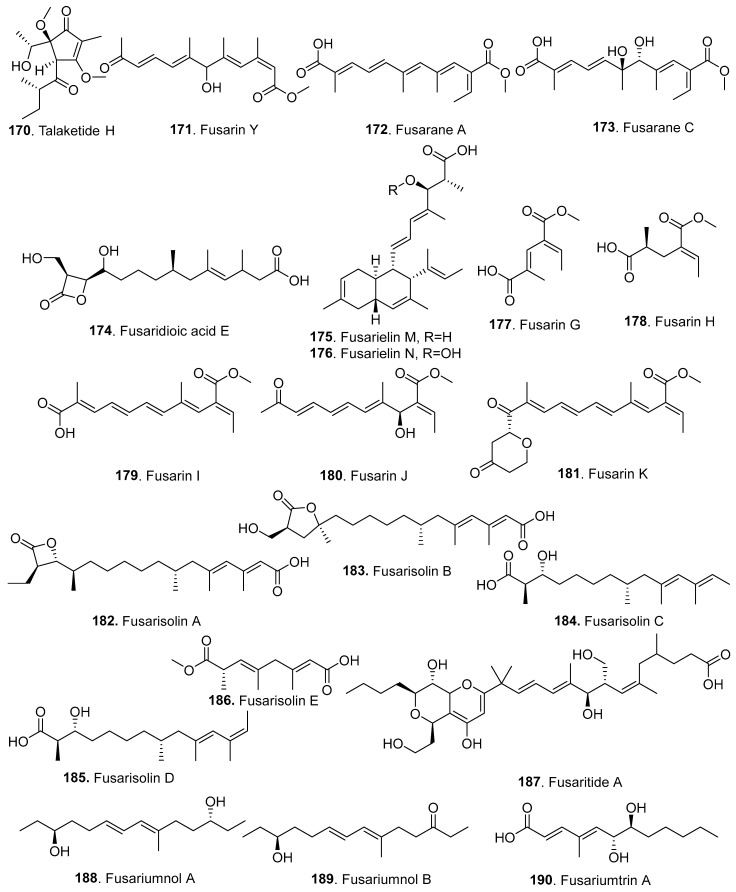

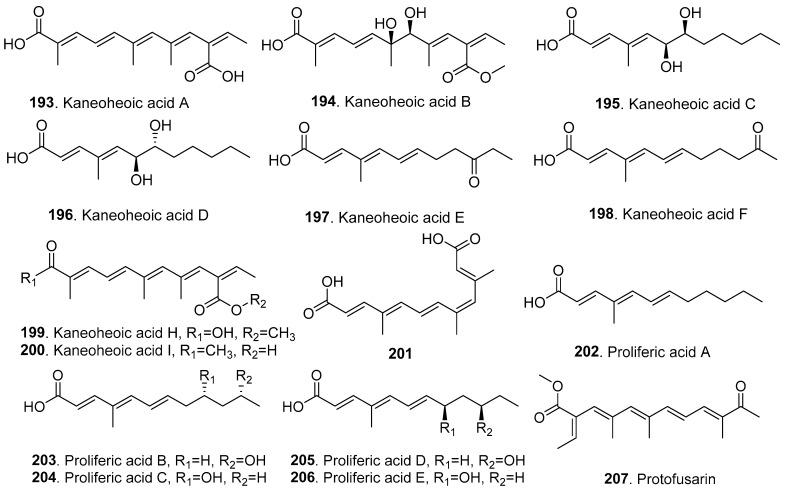
Structures of the other polyketides (**166**–**190** and **193**–**207**) isolated from *Fusarium* fungi.

Thirty-five other new polyketides (**166**–**207**) were isolated from *Fusarium* fungi, with their structures shown in Figure 13.

Five new polyketides, including asperpentenones B (**166**) and C (**167**), phomaligol J (**168**), and talaketides G (**169**) and H (**170**), were isolated from the mangrove-derived endophytic fungus *F. proliferatum* NSD-1 from *Kandelia candel* (Rhizophoraceae). Phomaligol J (**168**) and talaketides G (**169**) and H (**170**) showed cytotoxic activities, with IC_50_ values of 24.9 μM for A549 cells, 37.5 μM for SW480 cells, and 43.2 μM for A549 cells, respectively [86].

Fusarin Y (**171**) was a polyketide isolated from the *Fusarium* head blight pathogen *F. graminearum*. Fusarin Y (**171**) showed weak cytotoxic activity against three tumor cell lines [34].

Fusaranes A (**172**) and C (**173**) were isolated from cultures of *F. graminearum* [87,88]. Fusarane A (**172**) showed potent antibacterial activity against *Staphylococcus aureus* ATCC 29213, with an MIC value of 32 μg/mL [87].

Fusaridioic acid E (**174**) is an alkenoic acid analog isolated from the endophytic fungus *F. solani* MBM-5 of *Datura arborea* (Solanaceae). Fusaridioic acid E (**174**) exhibited significantly anti-inflammatory activity by inhibiting NO release from LPS-induced RAW264.7 cells, with an IC_50_ value of 77. 00 μM [89].

The secreted *Mycobacterium tuberculosis* protein tyrosine phosphatase B (MptpB) is an essential virulence factor required for the intracellular survival of *M. tuberculosis* within host macrophages. MptpB has become a promising target for developing novel anti-tuberculosis (TB) drugs. Two new fusarielins with a decalin core, namely, fusarielins M (**175**) and N (**176**), were isolated from the marine-derived fungus *F. graminearum* SYSU-MS5127, which was isolated from an anemone collected from Laishizhou Island in Shenzhen, China. Fusarielin M (**175**) was proved to be highly efficient in blocking MptpB-mediated Erk1/2 and p38 inactivation in macrophages and inhibiting mycobacterial growth within macrophages. In addition, fusarielin M (**175**) showed increased specificity for MptpB compared to MptpA and human PTP1B [90].

Fusarins G (**177**), H (**178**), I (**179**), J (**180**), and K (**181**) were isolated from the marine-derived fungus *F. solani* 7227, which was isolated from a seawater sample collected in the South China Sea. Among them, fusarins H (**178**), I (**179**), J (**180**), and K (**181**) showed anti-inflammatory activity by inhibiting lipopolysaccharide (LPS)-induced NO production in a murine macrophage cell line (RAW 264.7 cells) [33].

Five polyketides, fusarisolins A–E (**182**–**186**), were isolated from the marine-derived fungus *F. solani* H918, which was isolated from mangrove sediments. Fusarisolins A (**182**) and B (**183**) were the first two naturally occurring 21-carbon polyketides featuring a rare β- and γ-lactone unit, respectively. Fusarisolin A (**182**) significantly inhibited 3-hydroxy-3-methylglutaryl coenzyme A (HMG-CoA) synthase gene expression [91].

Fusaritide A (**187**) was isolated from the marine fish-derived halotolerant fungus *F. verticillioide* G102. It reduced cholesterol uptake by inhibiting Niemann–Pick C1-Like 1 (NPC1L1) [92].

Two novel aliphatic unsaturated alcohols, namely, fusariumnols A (**188**) and B (**189**), were isolated from the plant pathogenic fungus *F. proliferatum*, associated with diverse crops, including rice, wheat, maize, and garlic. Both compounds exhibited weak antibacterial activity against *Staphylococcus epidermidis*, with an MIC value of 100 μM [93].

Fusariumtrin A (**190**) was isolated from the entomogenous fungus *Fusarium* sp. LGWB-7 from *Harmonia axyridis* [54].

Gramiketides A (**191**) and B (**192**) were produced during the infection of wheat by *F. graminearum*. The molecular formulae of gramiketides A (**191**) and B (**192**) were C_29_H_44_O_8_ and C_29_H_42_O_7_, respectively, as estimated by LC-HRMS analysis. Unfortunately, their final structures were not determined [94].

Kaneoheoic acids A–F (**193**–**198**) were isolated from the marine-derived fungus *Fusarium* sp. FM701, which was isolated from a muddy sample from a Hawaiian beach [95].

Kaneoheoic acids H (**199**) and I (**200**) were isolated from the marine-derived fungus *F. graminearum* FM1010, which was isolated from shallow-water volcanic rock known as “live rock” at Richardson’s Beach, Hilo, Hawaii. Kaneoheoic acids H (**199**) and I (**200**) showed strong antibacterial activity against *Staphylococcus aureus* and methicillin-resistant *S. aureus*. In addition, kaneoheoic acid I (**200**) exhibited both anti-proliferative activity against ovarian cancer cell line A2780 and TNF-α-induced NF-κB inhibitory activity, with IC_50_ values of 18.52 and 15.86 μM, respectively [39].

One unnamed pentaene diacid analog, **201**, was isolated from the endophytic fungus *F. decemcellualre* F25, which was derived from the stems of the medicinal plant *Mahonia fortunei* (Berberidaceae) collected in Qindao, China [44].

Proliferic acids A (**202**), B (**203**), C (**204**), D (**205**), and E (**206**) were isolated from the fermentation cultures of the plant pathogenic fungus *F. proliferatum*. Among them, proliferic acid A (**202**) showed significant inhibitory activity against the growth of *Arabidopisis thaliana* roots. Proliferic acid A (**202**) was generated from proliferapyrone A (**122**) via the β-glucosidase-mediated hydrolysis of ester bonds and was inactivated by the intracellular oxidase-catalyzed oxidation of the terminal inert carbon atoms to form proliferic acids B (**203**), C (**204**), D (**205**), and E (**206**) [75].

Deleting a repressive histone methylation modification usually results in the derepression of secondary metabolite biosynthetic gene clusters (BGCs) in fungi. By using a mutant (Δ*Kmt6*) of *F. graminearum* lacking the H3K27 methyltransferase Kmt6, protofusarin (**207**) derived from the fusarin biosynthetic pathway was identified [96].

## 4. Terpenoids and Their Biological Activities

The terpenoids from *Fusarium* fungi mainly include sesquiterpenoids, diterpenoids, and triterpenoids. The terpenoids, their biological activities, *Fusarium* species, and their origins are shown in Table 3.

### 4.1. Sesquiterpenoids

**Figure 14:**
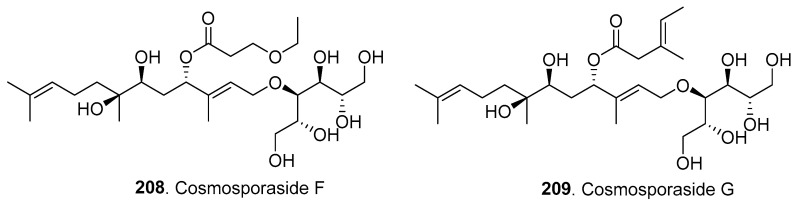

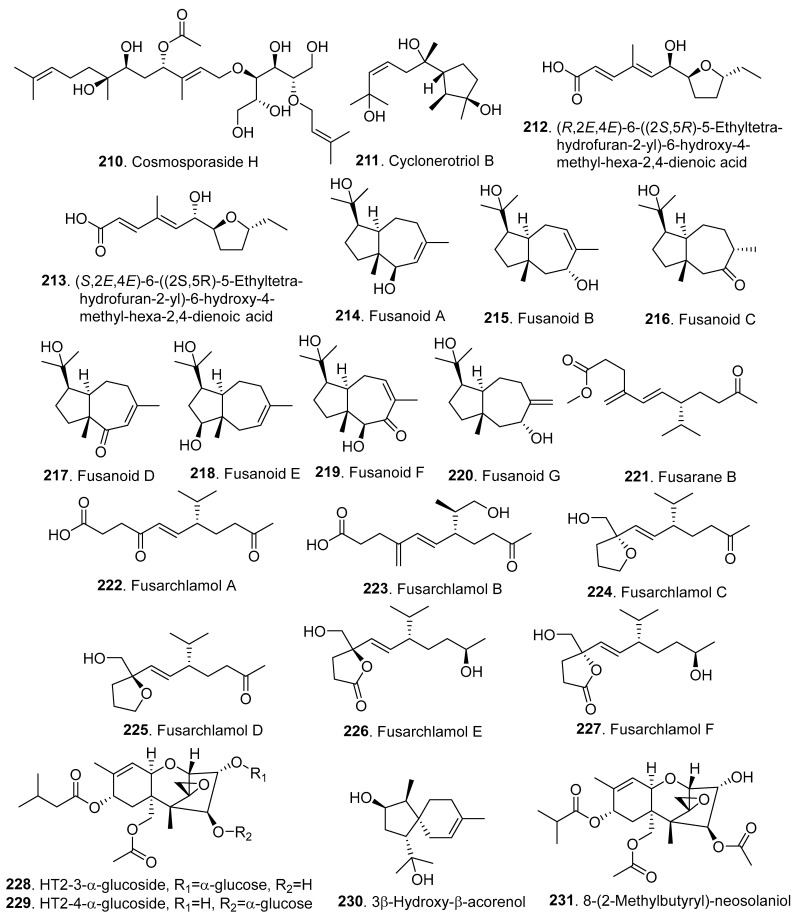

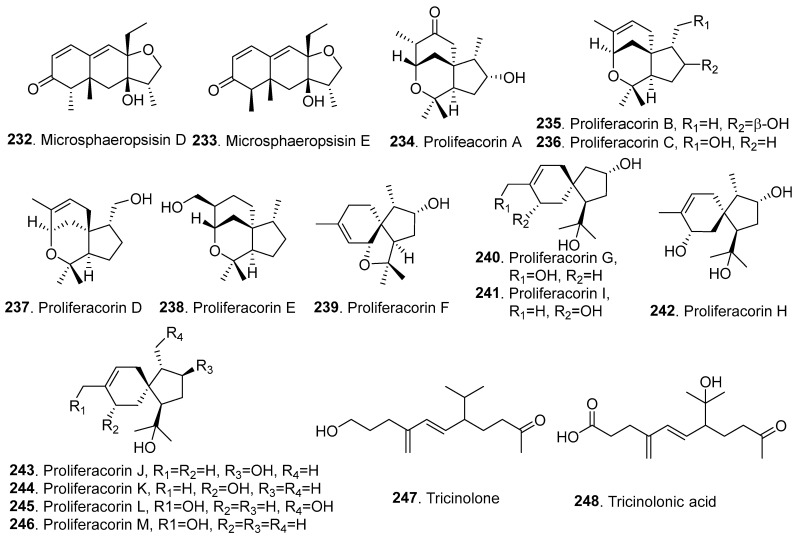
Structures of the sesquiterpenoids (**208**–**248**) isolated from *Fusarium* fungi.

Forty-one new sesquiterpenoids (**208**–**248**) were isolated from *Fusarium* fungi, and their structures are shown in Figure 14.

Three sugar-alcohol-conjugated acyclic sesquiterpenoids, namely, cosmosporasides F (**208**), G (**209**), and H (**210**), were isolated from the soil-derived fungus *F. oxysporum* SC0002, which was isolated from a soil sample collected in the Dinghu Mountain Biosphere Reserve located in Guangdong, China. Cosmosporasides F–H (**208**–**210**) showed weak antibacterial, cytotoxic, and anti-inflammatory activities at concentrations of 100 μg/mL, 10 μg/mL, and 50 μg/mL, respectively [97].

Cyclonerotriol B (**211**) is a cyclonerane sesquiterpenoid isolated from the soil-derived fungus *F. avenaceum* SF-1502 [64].

Two nor-sesquiterpenoids, namely, (*R*,2*E*,4*E*)-6-((2*S*,5*R*)-5-ethyltetrahydrofuran-2-yl)-6-hydroxy-4-methylhexa-2,4-dienoic acid (**212**) and (*S*,2*E*,4*E*)-6-((2*S*,5*R*)-5-ethyltetrahydrofuran-2-yl)-6-hydroxy-4-methylhexa-2,4-dienoic acid (**213**), were isolated from the endophytic fungus *F. tricinctum*, which was isolated from the roots of *Ligusticum chuanxiong* (Umbelliferae) collected in Sichuan, China. Of the two sesquiterpenoids, compound **212** exhibited moderate cytotoxic activity against MV4-11, with an IC_50_ value of 22.29 μM [98].

Seven carotane sesquiterpenoids named fusanoids A–G (**214**–**220**) were isolated from the fermentation broth of the desert plant endophytic fungus *Fusarium* sp. HM166, which was isolated from *Chenopodium quinoa* (Chenopodiaceae) collected from the Inner Mongolia Autonomous Region of West China. Among the compounds, fusanoid B (**215**) showed cytotoxic activity against MCF-7 cells of the human breast cancer cell line, while fusanoid D (**217**) showed potent inhibitory activity against IDH1^R132h^ mutant [99].

One sesquiterpenoid, namely, fusarane B (**221**), was isolated from cultures of the plant pathogenic fungus *F. graminearum*. Fusarane B (**221**) showed 61% and 75% inhibitory rates against HeLa cells and Mia PaCa2 cells, respectively, at 20 μM [88].

Six new sesquiterpenoids, fusarchlamols A–F (**222**–**227**), were isolated from an endophytic fungus, which was derived from sweet corn *Zea mays* (Gramnineae). Fusarchlamols A (**222**), B (**223**), E (**226**), and F (**227**) showed significant antifungal activity against the phytopathogen *Alternaria alternata* isolated from *Coffea arabica* (Rubiaceae), with an MIC value of 1 μg/mL [100].

Two HT2-glucosides, namely, HT2-3-*O*-α-glucoside (**228**) and HT2-4-*O*-α-glucoside (**229**), were isolated from rice cultures of *F. sporotrichioides*, the fungal pathogen of maize. The authors proposed that glucosyltransferase did not form both HT2-glucosides as they were in plants but by a *trans*-glycosylating α-glucosidase expressed by the fungus on the starch-containing rice medium [101].

3β-Hydroxy-β-acorenol (**230**) with an acorane framework was isolated from the endophytic fungus *F. proliferatum* AF-04, which was separated from the green Chinese onion (*Allium fistulosum*) [64].

8-(2-Methylbutyryl)-neosolaniol (**231**) was isolated from the plant endophytic fungus *F. sporotrichioides*, which was derived from the fruits of the medicinal plant *Rauvolfia yunnanensis* (Apocynaceae) collected in Yunnan, China [102].

Two new sesquiterpenoids, microsphaeropsisins D (**232**) and E (**233**), were isolated from the insect-derived fungus *F. lateritium* ZMT01. Microsphaeropsisin D (**232**) showed antifungal activities against *F. oxysporum*, *Penicillium italicum*, and *Colletotrichum musae*, with MIC values of 50, 25, and 25 mg/L, respectively. Microsphaeropsisin E (**233**) showed antifungal activities against *F. oxysporum*, *P. italicum*, *C. musae*, and *F. graminearum* with MICs of 25, 12.5, 12.5, and 100 mg/L, respectively. The in vivo bioassay showed that microsphaeropsisin E (**233**) displayed a control effect on banana anthracnose [103].

Thirteen new acorane sesquiterpenoids, namely, proliferacorins A–M (**234**–**246**), were isolated from the solid fermentation of the soil-derived *F. proliferatum*. Proliferacorins A–M (**234**–**246**) were tested for their cytotoxic, anti-inflammatory, and immunosuppressive activities. Nevertheless, none showed distinct inhibitory activity [104].

New molecules could be discovered by a combination of chemical and genetic dereplication. By using a double mutant (Δ*Kmt6*Δ*fus1*) of *F. graminearum*, two new sesquiterpenoids, tricinolone (**247**) and tricinolonoic acid (**248**), belonging to the tricindiol class were isolated [96].

### 4.2. Diterpenoids

**Figure 15:**
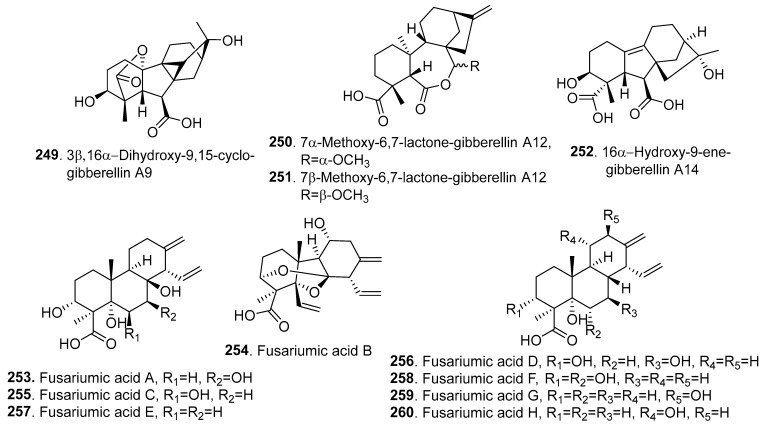
Structures of the diterpenoids (**249**–**260**) isolated from *Fusarium* fungi.

Twelve new diterpenoids (**249**–**260**) were isolated from *Fusarium* fungi, and their structures are shown in Figure 15.

Gibberellins (GAs) are well-known tetracyclic diterpenoid phytohormones. Four new GAs, namely, 3β,16α-dihydroxy-9,15-cyclo-gibberellin A9 (**249**), 7α-methoxy-6,7-lactone-gibberellin A12 (**250**), 7β-methoxy-6,7-lactone-gibberellin A12 (**251**), and 16α-hydroxy-9-ene-gibberellin A14 (**252**), were isolated from the endophytic fungus *Fusarium* sp. NJ-F5 from the roots of *Mahonia fortune* (Berberidaceae) collected in Nanjing, China. Among the isolated GAs, 3β,16α-dihydroxy-9,15-cyclo-gibberellin A9 (**249**) showed an obvious promoting effect on the seedling growth of *Arabidopsis thaliana* [105].

Eight isocassadiene-type diterpenoids, namely, fusariumic acids A–H (**253**–**260**), were isolated from the tomato *Fusarium* crown and root rot (FCRR) pathogen *F. oxysporum* f. sp. *radicis-lycopersici* (*Forl*). Among them, fusariumic acid B (**254**) contained a cage-like structure with an unusual 7,8-seco-isocassadiene skeleton. They all showed inhibitory activity against tomato seedlings at 200 μg/mL. Among them, fusariumic acid F (**258**) exhibited the strongest inhibition against hypocotyl and root elongation in tomato seedlings, with inhibitory rates of 61.3% and 45.3%, respectively [106].

### 4.3. Triterpenoids

**Figure 16:**
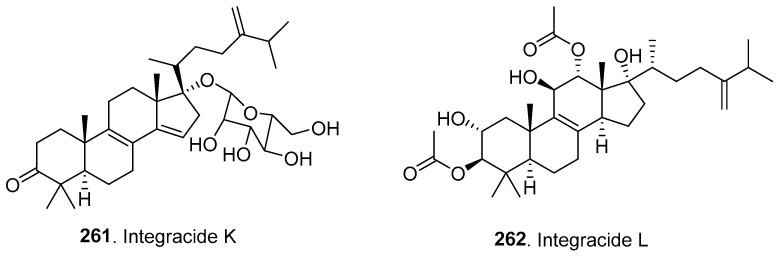
Structures of the triterpenoids (**261** and **262**) isolated from *Fusarium* fungi.

Two new triterpenoids (**261**, **262**) were isolated from *Fusarium* fungi, and their structures are shown in Figure 16.

Integracide K (**261**) was isolated from rice cultures of *F. armeniacum* M-3, an endophytic fungus from *Digitaria ciliaris* (Gramineae). Integracide K (**261**) exhibited moderate cytotoxicity against HeLa cells [107].

Integracide L (**262**) is a new tetracyclic triterpenoid isolated from the endophytic fungus *Fusarium* sp., which was separated from *Mentha longifolia* (Labiatae). Integracide L (**262**) showed strong 5-lipoxygenase inhibitory activity [108].

### 4.4. Other Terpenoids

**Figure 17:**
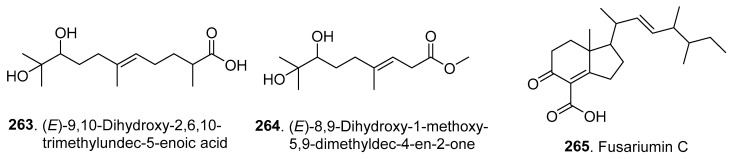
Structures of the other terpenoids (**263**–**265**) isolated from *Fusarium* fungi.

Three other new terpenoids (**263**–**265**) were isolated from *Fusarium* fungi, and their structures are shown in Figure 17.

Two chain terpenoids, namely, (*E*)-9,10-dihydroxy-2,6,10-trimethylundec-5-enoic acid (**263**) and (*E*)-8,9-dihydroxy-1-methoxy-5,9-dimethyldec-4-en-2-one (**264**), were isolated from the plant endophytic fungus *Fusarium* sp. HJT-P-2, which was isolated from *Rhodiola angusta* (Crassulaceae) from Changbai Mountain, Jilin Province, China [28].

Fusariumin C (**265**) is a meroterpene containing a cyclohexanone moiety isolated from *F. oxysporum* ZZP-R1, which was derived from the coastal plant *Rumex madio* Makino (Polygonaceae). Fusariumin C (**265**) displayed potent activity against *Staphyloccocus aureus*, with an MIC value of 6.25 μM [78].

## 5. Steroids and Their Biological Activities

**Figure 18:**
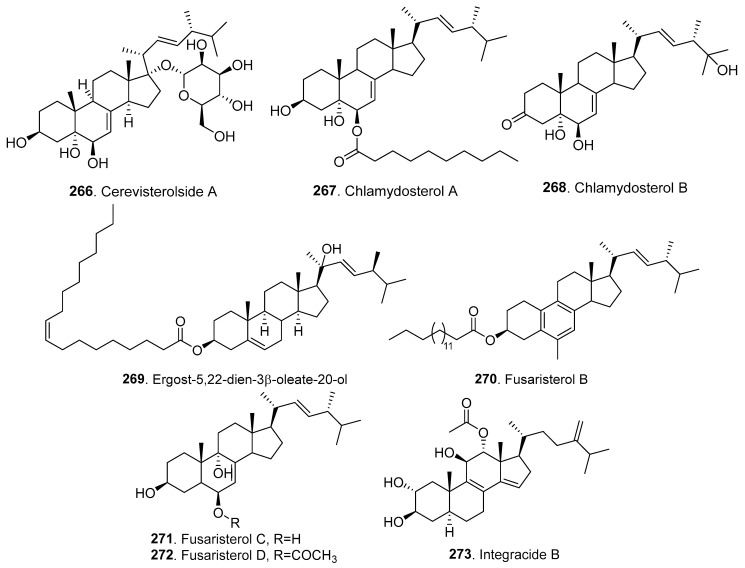
Structures of the steroids (**266**–**273**) isolated from *Fusarium* fungi.

The new steroids, their biological activities, *Fusarium* species, and their origins are shown in Table 4. The structures of 18 new steroids are shown in Figure 18.

Cerevisterolside A (**266**) was isolated from the mouse intestinal fungus *Fusarium* sp. LE06 of the murine cecum. Cerevisterolside A (**266**) showed significant growth inhibition against *Aspergillus fumigatus*, *F. oxysporum*, and *Verticillium dahlia*, with MIC values in the range of 1.56–6.25 μg/mL [72].

Two new ergosterol derivatives, namely, chlamydosterols A (**267**) and B (**268**), were obtained from the endophytic fungus *F. chlamydosporum*, which was isolated from the leaves of *Anvillea garcinii* (Asteraceae) growing in Saudi Arabia. Chlamydosterol A (**267**) displayed anti-inflammatory activity by inhibiting 5-lipooxygenase (5-LOX) activity with an IC_50_ value of 3.06 μM [109].

One new ergostane-type sterol, namely, ergost-5,22*E*-dien-3*β*-oleate-20-ol (**269**), was isolated from the endophytic fungus *F. phaeoli*, which was isolated from the roots of *Chisocheton macrophyllus* (Meliaceae) [110].

Three ergosterol derivatives, namely, fusaristerols B (**270**), C (**271**), and D (**272**), were isolated from the endophytic fungus *Fusarium* sp. isolated from *Mentha longifolia* (Labiatae) roots growing in Saudi Arabia. Fusaristerols B (**270**) and C (**271**) showed anti-inflammatory activity by exhibiting 5-LOX inhibitory capacity, with IC_50_ values of 3.61 and 2.45 μM, respectively. The structure–activity relationship indicated that the absence of the chain fatty acyl moiety and peroxy group dramatically decreased anti-inflammatory activity [111].

Integracide B (**273**) was isolated from the rice cultures of *F. armeniacum* M-3, an endophytic fungus from *Digitaria ciliaris* (Gramineae) [107].

## 6. Phenolics and Their Biological Activities

**Figure 19:**
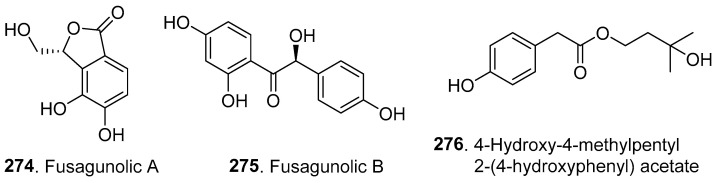
Structures of the phenolic metabolites (**274**–**276**) isolated from *Fusarium* fungi.

The phenolics, their biological activities, *Fusarium* species, and their origins are shown in Table 5. The structures of three new phenolics are shown in Figure 19.

Two new phenolic compounds, fusagunolics A (**274**) and B (**275**), were isolated from the plant endophytic fungus *F. guttiforme*. Fusagunolic B (**275**) showed moderate anti-inflammatory activity by inhibiting nitric oxide (NO) production with an IC_50_ value of 28.6 μM [112].

4-Hydroxy-4-methylpentyl 2-(4-hydroxyphenyl) acetate (**276**) is a phenolic compound isolated from the endophytic fungus *Fusarium* sp. HJT-P-2 of *Rhodiola serrata* (Crassulaceae). The compound showed weak inhibitory effects on the root growth of *Arabidopsis thaliana* [113].

## 7. Conclusions and Perspectives

In this mini-review, we summarized the chemical structures, biological activities, occurrence, and fungal origin of 276 newly isolated secondary metabolites from at least 21 *Fusarium* species reported from 2019 to October 2024 (Table S1). The main secondary metabolites belong to nitrogen-containing metabolites, polyketides, terpenoids, steroids, and phenolics. Some metabolites exhibited obvious biological activities.

Some *Fusarium* secondary metabolites, such as imidazole alkaloids, including fusaritricines A–I (**78**–**86**), (+)-fusaritricine J (**87**), (−)-fusaritricine J (**88**), and fusaritricines K–P (**89**–**94**), were isolated from *F. tricinctum* [62,63]. These imidazole alkaloids were also only isolated from the genus *Fusarium*. These metabolites may display chemotaxonomic significance, which should be further studied.

Some *Fusarium* metabolites, such as apicidin L (**40**), as an antimalarial [47]; beauvericin H (**41**), as an anti-tumor agent [48]; and chlamydosterol A (**267**), as an anti-inflammatory agent [109], have shown their potential medicinal and agricultural applications [114,115]. Some *Fusarium*-derived phytotoxins could be used as fungal herbicide candidates [21,27]. The endophytic *F. oxysprum* strains could be used as biocontrol agents against other pathogenic fungi such as *Verticillium dahlia*, *Pythium ultimum*, and *Botrytis cinerea* with their complex biocontrol mechanisms [115].

Many biosynthetic pathways that link secondary metabolites with their corresponding BGCs have been identified in *Fusarium* fungi. Therefore, most of these metabolites linked to their BGCs must be investigated [20]. In order to search for new bioactive metabolites from *Fusarium* fungi, some strategies, such as the one strain many compounds (OSMAC) approach [116], environmental factor regulation [117], transcriptional regulation [19], epigenetic regulation [118], gene deletion and overexpression [52,96], co-cultivation [119], and heterologous expression [120], have been proven to be effective to activate the silent gene clusters and produce more secondary metabolites in *Fusasrium* fungi.

In the past, *Fusarium* secondary metabolites were mainly obtained from plant pathogenic *Fusarium* species. The secondary metabolites usually belonged to mycotoxins or phytotoxins such as beauvericin, enniatins, fumonisins, fusaproliferin, fusaric acid, moniliformin, trichothecenes, and zearalenone [2,17,121]. However, in recent years, more and more secondary metabolites were isolated from plant endophytic [5], soil-derived [64,97], and marine-derived *Fusarium* species [122]. In addition to phytotoxic and animal toxic activities, the biological activities of *Fusarum* metabolites are also manifested in many other aspects such as anti-virus, antimalarial, anti-inflammatory, and neuroprotective activities, as well as the inhibitory activities toward enzymes (Table 1, Table 2, Table 3, Table 4 and Table 5).

Though major *Fusarium* species have been studied for their metabolites [5], the remaining fungal species in the *Fusarium* genus need to be revealed in detail. Moreover, the biological activities, structure–activity relationships, mechanisms of action, biosynthesis, and biosynthesis regulation of the metabolites of *Fusarium* fungi need to be further investigated. The clarification of the metabolites of *Fusarium* fungi may not only promote the discovery of more compounds with novel structures and excellent biological activities but also provide a better understanding the physiological, ecological, and chemotaxonomic significance of *Fusarium* fungi.

## Figures and Tables

**Table 1:** New nitrogen-containing metabolites (**1**–**97**) isolated from *Fusarium* fungi and their biological activities.

Metabolite Class	Metabolite Name	Biological Activity	*Fusarium* Species	Fungal Origin	Ref.
Amines					
	2-Amino-14,16-dimethyloctadecan-3-ol (**1**)	Cytotoxic activity	*F. avenaceum*	Plant pathogenic fungus	[24]
	Deacetyl fusarochromene (**2**); 4′-*O*-acetyl fusarochromanone (**3**)	Antimalarial activity	*Fusarium* sp.	Entomogenous fungus	[25]
	Deacetamidofusarochrom-2′,3′-diene (**4**)	Cytotoxic and antibacterial activities	*F. equiseti*	Marine-derived fungus	[26]
Amides					
	Decalintertracids A (**5/6**) and B (**7**/**8**)	Phytotoxic activity	*F. equiseti*	Plant endophytic fungus	[27]
	(3*E*,7*E*)-11,12-Dihydroxy-4,8,12-trimethyltrideca-3,7-dienamide (**9**)	-	*Fusarium* sp.	Plant endophytic fungus	[28]
	(*S*,*E*)-Methyl-2-(2,4-dimethylhex-2-enamido)acetate (**10**)	-	*F. oxysporum*	Plant endophytic fungus	[29]
	DihydroNG393 (**11**); dihydrolucilactaene (**12**); 13α-hydroxylucilactaene (**13**)	Antimalarial activity	*Fusarium* sp.	Plant endophytic fungus	[30]
	Fusaindoterpene A (**14**)	-	*Fusarium* sp.	Marine-derived fungus	[31]
	Fusaramin (**15**)	Antibacterial and antimitochondrial activities	*Fusarium* sp.	Soil-derived fungus	[32]
	Fusarin L (**16**)	Anti-inflammatory activity	*F. solani*	Marine-derived fungus	[33]
	Furarin X1 (**17**)	Cytotoxic activity	*F. graminearum*	Plant pathogenic fungus	[34]
	Fusarisetin B (**18**)	Cytotoxic activity	*F. equiseti*	Entomogenous fungus	[35]
	Fusarisetins C (**19**) and D (**20**)	-	*F. equiseti*	Marine-derived fungus	[36]
	Fusaribenzamide A (**21**)	Antifungal activity	*Fusarium* sp.	Plant endophytic fungus	[37]
	Fusarochromene (**22**)	-	*F. sacchari*	Plant pathogenic fungus	[38]
	Kaneoheoic acid G (**23**)	Antibacterial activity	*F. graminearum*	Marine-derived fungus	[39]
	8(*Z*)-Lucilactaene (**24**); 4(*Z*)-lucilactaene (**25**)	Anti-inflammatory activity	*Fusarium* sp.	Plant endophytic fungus	[40]
	*N*-({4-[(3-methylbut-2-en-1-yl)oxy]phenyl}acetyl)glycine (**26**); methyl *N*-({4-[(3-methylbut-2-en-1-yl)oxy]phenyl}acetyl)glycinate (**27**)	-	*Fusarium* sp.	Marine-derived fungus	[41]
	Prelucilactaenes G (**28**) and H (**29**)	Antimalarial activity	*Fusarium* sp.	Plant endophytic fungus	[42]
	Proliferatins A–C (**30**–**32**)	Anti-inflammatory activity	*F. proliferatum*	Fungal stroma-derived fungus	[43]
	Pyrrolidinone analogs **33**, **34**, and **35**	-	*F. decemcellulare*	Plant endophytic fungus	[44]
Cyclic peptides					
	Acuminatums E (**36**) and F (**37**)	Antifungal activity	*F. lateritium*	Plant endophytic fungus	[45]
	Amoenamide C (**38**); sclerotiamide B (**39**)	Antimicrobial and larvicidal activities	*F. sambucinum*	Plant endophytic fungus	[46]
	Apicidin L (**40**)	Cytotoxic and antimalarial activities	*F. fujikuroi*	Plant pathogenic fungus	[47]
	Beauvervin H (**41**)	Cytotoxic activity	*Fusarium* sp.	Plant endophytic fungus	[48]
	Beauvericins M (**42**) and N (**43**)	-	*Fusarium* sp.	Plant endophytic fungus	[49]
	Cyclo-(L-Trp-L-Phe-L-Phe) (**44**)	Cytotoxic and antibacterial activities	*F. proliferatum*	Plant endophytic fungus	[50]
	Enniatin W (**45**)	Cytotoxic activity	*F. oxysporum*	Plant endophytic fungus	[51]
	Fusahexin (**46**)	-	*F. graminearum*	Plant pathogenic fungus	[52]
	Fusaristatins D–F (**47**–**49**)	-	*Fusarium* sp.	Plant endophytic fungus	[53]
	Gramipiperazines A (**50**) and B (**51**)	Antibacterial activity	*F. graminearum*	Marine-derived fungus	[39]
Pyridines					
	Climacomontaninate D (**52**)	-	*Fusarium* sp.	Entomogenous fungus	[54]
	Fusaricates H–K (**53**–**56**)	-	*F. solani*	Mangrove-derived fungus	[55]
	Fasaripyridines A (**57**) and B (**58**)	Antimicrobial and cytotoxic activities	*Fusarium* sp.	Marine-derived fungus	[56]
Pyridones					
	Fusapyridons C (**59**) and D (**60**)	Cytotoxic activity	*F. avenaceum*	Entomopathogenic fungus	[57]
	Fusarone A (**61**)	Cytotoxic and antibacterial activities	*F. proliferatum*	Plant endophytic fungus	[50]
	1′-Methoxy-6′-*epi*-oxysporidinone (**62**)	-	*F. concentricum*	Plant endophytic fungus	[58]
Indole analogs					
	Chlamydosporin (**63**)	Phytotoxic activity	*F. chlamydosporum*	Plant endophytic fungus	[59]
	Ethyl 3-indoleacetate (**64**)	Cytotoxic and antibacterial activities	*F. proliferatum*	Plant endophytic fungus	[50]
	Fusaconate A (**65**)	-	*F. concentricum*	Plant endophytic fungus	[58]
	Fusarindoles A–E (**66**–**70**)	-	*F. equiseti*	Marine-derived fungus	[60]
	(+)-Fusaspoid A (**71**); (−)-fusaspoid A (**72**)	-	*Fusarium* sp.	Marine-derived fungus	[61]
	Fusaindoterpene B (**73**); fusarindoles A–C (**74**–**76**); isoalternatine A (**77**)	Inhibitory activity against Zika virus	*Fusarium* sp.	Marine-derived fungus	[31]
Imidazole analogs					
	Fusaritricines A–I (**78**–**86**)	Antibacterial activity	*F. tricinctum*	Plant endophytic fungus	[62]
	(+)-Fusaritricine J (**87**), (−)-fusaritricine J (**88**), and fusaritricines K–P (**89**–**94**)	Antibacterial activity	*F. tricinctum*	Plant endophytic fungus	[63]
Others					
	Fusaravenin (**95**)	-	*F. avenaceum*	Soil-derived fungus	[64]
	Fusaroxazin (**96**)	Cytotoxic and antimicrobial activities	*F. oxysporum*	Plant endophytic fungus	[65]
	Secobeauvericin A (**97**)	-	*Fusarium* sp.	Plant endophytic fungus	[49]

**Table 2:** New polyketides (**98**–**207**) isolated from *Fusarium* fungi and their biological activities.

Metabolite Class	Metabolite Name	Biological Activity	*Fusarium* Species	Fungal Origin	Ref.
Pyrones: α-pyrones					
	(7*S*,8*R*)-Chlamydospordiol (**98**)	-	*Fusarium* sp.	Plant endophytic fungus	[53]
	6-((2*S*,3*S*)-2,3-Dihydroxybutan-2-yl)-3-methyl-2*H*-pyran-2-one (**99**); 6-((2*R*,3*R*)-2,3-dihydroxybutan-2-yl)-3-methyl-2*H*-pyran-2-one (**100**); 6-((2*S*,3*R*)-2,3-dihydroxybutan-2-yl)-3-methyl-2*H*-pyran-2-one (**101**); 6-((2*R*,3*S*)-2,3-dihydroxybutan-2-yl)-3-methyl-2*H*-pyran-2-one (**102**)	Cytotoxic activity	*F. tricinctum*	Plant endophytic fungus	[66]
	Dihydrolateropyrone (**103**)	-	*F. tricinctum*	Plant endophytic fungus	[67]
	Fupyrones A (**104**) and B (**105**)	-	*Fusarium* sp.	Plant endophytic fungus	[68]
	Fusaisocoumarin A (**106**)	Antifungal activity	*F. verticillioides*	Plant endophytic fungus	[69]
	Fusaripyrones C (**107**) and D (**108**)	-	*Fusarium* sp.	Plant endophytic fungus	[70]
	Fusaritricins A (**109**/**110**) and B–D (**111**–**113**)	Antibacterial activity	*F. tricinctum*	Plant endophytic fungus	[71]
	Fusintespyrone A (**114**)	Antifungal activity	*Fusarium* sp.	Intestinal fungus	[72]
	Fusopoltides B (**115**) and C (**116**)	-	*F. solani*	Plant endophytic fungus	[73]
	7-Hydroxy-3-(2-hydroxy-propyl)-5-methyl-*epi*-isochromen-1-one (**117**)	-	*F. graminearum*	Marine-derived fungus	[39]
	Isocoumarin analogs **118**, **119**, and **120**	-	*F. decemcellulare*	Plant endophytic fungus	[44]
	Karimunone A (**121**)	Antibacterial activity	*Fusarium* sp.	Marine-derived fungus	[74]
	Proliferapyrone A (**122**)	-	*F. proliferatum*	Plant pathogenic fungus	[75]
Pyrones: γ-pyrones					
	Fusapyrone A (**123**)	Cytotoxic activity	*Fusarium* sp.	Desert-derived fungus	[76]
	Fusaresters A–E (**124**–**128**)	Inhibitory activity against protein tyrosine phosphatase	*Fusarium* sp.	Marine-derived fungus	[77]
	Fusariumin D (**129**)	Antibacterial activity	*F. oxysporum*	Plant endophytic fungus	[77,78]
	Fusolanonones A (**130**) and B (**131**)	Antibacterial activity	*F. solani*	Mangrove-derived fungus	[55]
	6-((9*R*,11*R*,*E*)-13-Hydroxy-9,11-dimethyloct-7-en-7-yl)-2-methoxy-4*H*-pyran-4-one (**132**)	Neuroprotective activity	*F. solani*	Plant endophytic fungus	[79]
Furanones					
	Curvicollides E–G (**133**–**135**), Ha (**136**), and Hb (**137**)	Cytotoxic activity	*F. armeniacum*	Plant endophytic fungus	[80]
	Fusarisetin B (**138**)	Cytotoxic activity	*F. equiseti*	Entomogenous fungus	[35]
	Fusaspirols A–D (**139**–**142**)	Osteoclastic differentiation activity	*F. solani*	Plant endophytic fungus	[73]
	Fusopoltides D (**143**) and E (**144**)	-	*F. solani*	Plant endophytic fungus	[81]
	Neovasifuranones A (**145**) and B (**146**)	Antibacterial activity	*F. oxysporum*	Plant endophytic fungus	[82]
	Spiroleptosphols T1 (**147**), T2 (**148**), and U–Z (**149**–**154**)	-	*F. avenaceum*	Plant endophytic fungus	[83]
Quinones					
	6-Hydroxiy-astropaquinone B (**155**); astropaquinone D (**156**)	Antibacterial and phytotoxic activities	*F. napiforme*	Mangrove-derived fungus	[84]
	1-Methoxylfusarnaphthoquinone A (**157**); 1-dehydroxysolaninol (**158**); 5-dehydroxysolaninol (**159**); fusarnaphthoquinone D (**160**)	Cytotoxic activity	*Fusarium* sp.	Plant endophytic fungus	[85]
	Fusatricinones A–D (**161**–**164**)	-	*F. tricinctum*	Plant endophytic fungus	[67]
	Karimunone A (**165**)	-	*Fusarium* sp.	Marine-derived fungus	[74]
Others					
	Asperpentenones B (**166**) and C (**167**); phomaligol J (**168**); talaketides G (**169**) and H (**170**)	Cytotoxic activity	*F. proliferatum*	Mangrove-derived fungus	[86]
	Furarin Y (**171**)	Cytotoxic activity	*F. graminearum*	Plant pathogenic fungus	[34]
	Fusaranes A (**172**) and C (**173**)	Antibacterial activity	*F. graminearum*	Plant pathogenic fungus	[87,88]
	Fusaridioic acid E (**174**)	Anti-inflammatory activity	*F. solani*	Plant endophytic fungus	[89]
	Fusarielins M (**175**) and N (**176**)	Inhibitory activity against protein tyrosine phosphatase B	*F. graminearum*	Marine-derived fungus	[90]
	Fusarins G–K (**177**–**181**)	Anti-inflammatory activity	*F. solani*	Marine-derived fungus	[33]
	Fusarisolins A–E (**182**–**186**)	Inhibition of HMG-CoA synthase gene expression	*F. solani*	Marine-derived fungus	[91]
	Fusaritide A (**187**)	Reduced cholesterol uptake	*F. verticillioide*	Marine fish-derived fungus	[92]
	Fusariumnols A (**188**) and B (**189**)	Antibacterial activity	*F. proliferatum*	Plant pathogenic fungus	[93]
	Fusariumtrin A (**190**)	-	*Fusarium* sp.	Entomogenous fungus	[54]
	Gramiketides A (**191**) and B (**192**)	-	*F. graminearum*	Plant pathogenic fungus	[94]
	Kaneoheoic acids A–F (**193**–**198**)	-	*Fusarium* sp.	Marine-derived fungus	[95]
	Kaneoheoic acids H (**199**) and I (**200**)	Antibacterial and cytotoxic activities	*F. graminearum*	Marine-derived fungus	[39]
	Pentaene diacid analog **201**	-	*F. decemcellulare*	Plant endophytic fungus	[44]
	Proliferic acids A–E (**202**–**206**)	Phytotoxic activity	*F. proliferatum*	Plant pathogenic fungus	[75]
	Protofusarin (**207**)	-	*F. graminearum*	Plant pathogenic fungus	[96]

**Table 3:** New terpenoids (**208**–**265**) isolated from *Fusarium* fungi and their biological activities.

Metabolite Class	Metabolite Name	Biological Activity	*Fusarium* Species	Fungal Origin	Ref.
Sesquiterpenoids					
	Cosmosporasides F–H (**208**–**210**)	Antibacterial, cytotoxic, and anti-inflammatory activities	*F. oxysporum*	Soil-derived fungus	[97]
	Cyclonerotriol B (**211**)	-	*F. avenaceum*	Soil-derived fungus	[64]
	(*R*,2*E*,4*E*)-6-((2*S*,5*R*)-5-Ethyltetrahydrofuran-2-yl)-6-hydroxy-4-methylhexa-2,4-dienoic acid (**212**); (*S*,2*E*,4*E*)-6-((2*S*,5*R*)-5-ethyltetrahydrofuran-2-yl)-6-hydroxy-4-methylhexa-2,4-dienoic acid (**213**)	Cytotoxic activity	*F. tricinctum*	Plant endophytic fungus	[98]
	Fusanoids A–G (**214**–**220**)	Cytotoxic activity	*Fusarium* sp.	Plant endophytic fungus	[99]
	Fusarane B (**221**)	Cytotoxic activity	*F. graminearum*	Plant pathogenic fungus	[88]
	Fusarchlamols A–F (**222**–**227**)	Antifungal activity	*Fusarium* sp.	Plant endophytic fungus	[100]
	HT2-3-*O*-α-glucoside (**228**); HT2-4-*O*-α-glucoside (**229**)	-	*F. sporotrichioides*	Plant pathogenic fungus	[101]
	3β-Hydroxy-β-acorenol (**230**)	-	*F. proliferatum*	Plant endophytic fungus	[64]
	8-(2-Methylbutyryl)-neosolaniol (**231**)	-	*F. sporotrichioides*	Plant endophytic fungus	[102]
	Microsphaeropsisins D (**232**) and E (**233**)	Antifungal activity	*F. lateritium*	Insect-derived fungus	[103]
	Proliferacorins A–M (**234**–**246**)	-	*F. proliferatum*	Soil-derived fungus	[104]
	Tricinolone (**247**); tricinolonoic acid (**248**)	-	*F. graminearum*	Plant pathogenic fungus	[96]
Diterpenoids					
	3β,16α-Dihydroxy-9,15-cyclo-gibberellin A9 (**249**); 7α-methoxy-6,7-lactone-gibberellin A_12_ (**250**); 7β-methoxy-6,7-lactone-gibberellin A12 (**251**); 16α-hydroxy-9-ene-gibberellin A14 (**252**)	Promoting effect on seedling growth	*Fusarium* sp.	Plant endophytic fungus	[105]
	Fusarium acids A–H (**253**–**260**)	Inhibition of hypocotyl and root elongation in tomato seedlings	*F. oxysporum* f.sp*. radicis-lycopersici*	Plant pathogenic fungus	[106]
Triterpenoids					
	Integracide K (**261**)	Cytotoxic activity	*F. armeniacum*	Plant endophytic fungus	[107]
	Integracide L (**262**)	Lipoxygenase inhibitory activity	*Fusarium* sp.	Plant endophytic fungus	[108]
Others					
	(*E*)-9,10-Dihydroxy-2,6,10-trimethylundec-5-enoic acid (**263**); (*E*)-8,9-dihydroxy-1-methoxy-5,9-dimethyldec-4-en-2-one (**264**)	-	*Fusarium* sp.	Plant endophytic fungus	[28]
	Fusariumin C (**265**)	Antibacterial activity	*F. oxysporum*	Plant endophytic fungus	[78]

**Table 4:** New steroids (**266**–**273**) isolated from *Fusarium* fungi and their biological activities.

Metabolite Name	Biological Activity	*Fusarium* Species	Fungal Origin	Ref.
Cerevisterolside A (**266**)	Antifungal activity	*Fusarium* sp.	Mouse intestinal fungus	[72]
Chlamydosterols A (**267**) and B (**268**)	Anti-inflammatory activity	*F. chlamydosporum*	Plant endophytic fungus	[109]
Ergost-5,22*E*-dien-3*β*-oleate-20-ol (**269**)	-	*F. phaeoli*	Plant endophytic fungus	[110]
Fusaristerols B–D (**270**–**272**)	Anti-inflammatory activity	*Fusarium* sp.	Plant endophytic fungus	[111]
Integracide B (**273**)	-	*F. armeniacum*	Plant endophytic fungus	[107]

**Table 5:** New phenolic metabolites (**274**–**276**) isolated from *Fusarium* fungi and their biological activities.

Metabolite Name	Biological Activity	*Fusarium* Species	Fungal Origin	Ref.
Fusagunolics A (**274**) and B (**275**)	Anti-inflammatory activity	*F. guttiforme*	Plant endophytic fungus	[112]
4-Hydroxy-4-methylpentyl 2-(4-hydroxyphenyl) acetate (**276**)	Phytotoxic activity	*Fusarium* sp.	Plant endophytic fungus	[113]

## Supplementary Materials

The following supporting information can be downloaded at: https://www.mdpi.com/article/10.3390/jof10110778/s1, Table S1: List of *Fusarium* species used for the isolation of new secondary metabolites reported from 2019 to October 2024. All the references cited in Supplementary Table S1 are listed in the References section of the text.
