# Supplementary material for: Recent Updates on the Secondary Metabolites from Fusarium Fungi and Their Biological Activities (Covering 2019 to 2024)
Source: J Fungi (Basel). 2024 Nov 9;10(11):778. doi: 10.3390/jof10110778 (PMC11596042; doi:10.3390/jof10110778)
Supplement: Supplementary file 1 [file jof-10-00778-s001.zip › jof-3270040-supplementary.pdf]

Supplementary Materials:

Recent Updates on the Secondary Metabolites from *Fusarium* Fungi and Their Biological Activities (Covering 2019 to 2024)

Prosper Amuzu, Xiaoqian Pan, Xuwen Hou, Jiahang Sun, Muhammad Abubakar Jakada, Eromosele Odigie, Dan Xu, Daowan Lai, and Ligang Zhou *

Department of Plant Pathology and MOA Key Lab of Pest Monitoring and Green Management, College of Plant Protection, China Agricultural University, Beijing 100193, China; amuzuprosper07@cau.edu.cn (P.A.); xiaoqianpan@cau.edu.cn (X.P.); xwhou@cau.edu.cn (X.H.); jiahangsun@cau.edu.cn (J.S.); jakada@cau.edu.cn (M.A.J.); eromoseleodigie@cau.edu.cn (E.O.); cauxudan@cau.edu.cn (D.X.); dwlai@cau.edu.cn (D.L.)
* Correspondence: lgzhou@cau.edu.cn (L.Z.); Tel.: +86-10-6273-1199

Table S1. The list of *Fusarium* species used for isolation of new secondary metabolites reported from 2019 to October 2024.

Fusarium Species	Fungal Origin	Metabolite Name	Metabolite Class	Biological Activity	Ref.
Fusarium armeniacum	Plant endophytic fungus	Curvicollides E–G (**133–135**), Ha (**136**) and Hb (**137**)	Polyketides: furanones	Cytotoxic activity	[80]
Fusarium armeniacum	Plant endophytic fungus	Integracide K (**261**)	Terpenoids: triterpenoids	Cytotoxic activity	[107]
Fusarium armeniacum	Plant endophytic fungus	Integracide B (**273**)	Steroids	-	[107]
Fusarium avenaceum	Plant pathogenic fungus	2-Amino-14,16-dimethyloctadecan-3-ol (**1**)	Nitrogen-containing: amines	Cytotoxic activity	[24]
Fusarium avenaceum	Entomopathogenic fungus	Fusapyridons C (**59**) and D (**60**)	Nitrogen-containing: pyridones	Cytotoxic activity	[57]
Fusarium avenaceum	Soil-derived fungus	Fusaravenin (**95**)	Nitrogen-containing: others	-	[64]
Fusarium avenaceum	Plant endophytic fungus	Spiroleptosphols T1 (**147**) and T2 (**148**), and U–Z (**149–154**)	Polyketides: furanones	-	[83]
Fusarium avenaceum	Soil-derived fungus	Cyclonerotriol B (**211**)	Terpenoids: sesquiterpenoids	-	[64]
Fusarium chlamydosporum	Plant endophytic fungus	Chlamydosporin (**63**)	Nitrogen-containing: indoles	Phytotoxic activity	[59]
Fusarium chlamydosporum	Plant endophytic fungus	Chlamydosterols A (**267**) and B (**268**)	Steroids	Anti-inflammatory activity	[109]
Fusarium concentricum	Plant endophytic fungus	1′-Methoxy-6′-*epi*-oxysporidinone (**62**)	Nitrogen-containing: pyridones	-	[58]
Fusarium concentricum	Plant endophytic fungus	Fusaconate A (**65**)	Nitrogen-containing: indoles	-	[58]
Fusarium decemcellulare	Plant endophytic fungus	Pyrrolidinone analogs **33**, **34**, and **35**	Nitrogen-containing: amides	-	[44]
Fusarium decemcellulare	Plant endophytic fungus	Isocoumarin analogs **118**, **119**, and **120**	Polyketides: α-pyrones	-	[44]
Fusarium decemcellulare	Plant endophytic fungus	Pentaene diacid analog **201**	Polyketides: others	-	[44]

Fusarium equiseti	Marine-derived fungus	Deacetamidofusarochrom-2′,3′-diene (**4**)	Nitrogen-containing: amines	Cytotoxic and antibacterial activities	[26]
Fusarium equiseti	Plant endophytic fungus	Decalintertracids A (**5/6**) and B (**7/8**)	Nitrogen-containing: amides	Phytotoxic activity	[27]
Fusarium equiseti	Entomogenous fungus	Fusarisetin B (**18**)	Nitrogen-containing: amides	Cytotoxic activity	[35]
Fusarium equiseti	Marine-derived fungus	Fusarisetins C (**19**) and D (**20**)	Nitrogen-containing: amides	-	[36]
Fusarium equiseti	Marine-derived fungus	Fusarindoles A–E (**66–70**)	Nitrogen-containing: indoles	-	[60]
Fusarium equiseti	Entomogenous fungus	Fusarisetin B (**138**)	Polyketides: furanones	Cytotoxic activity	[35]
Fusarium fujikuroi	Plant pathogenic fungus	Apicidin L (**40**)	Nitrogen-containing: cyclic peptides	Cytotoxic and antimalarial activity.	[47]
Fusarium graminearum	Plant pathogenic fungus	Furarin X1 (**17**)	Nitrogen-containing: amides	Cytotoxic activity	[34]
Fusarium graminearum	Marine-derived fungus	Kaneoheoic acid G (**23**)	Nitrogen-containing: amides	Antibacterial activity	[39]
Fusarium graminearum	Plant pathogenic fungus	Fusahexin (**46**)	Nitrogen-containing: cyclic peptides	-	[52]
Fusarium graminearum	Marine-derived fungus	Gramipiperazines A (**50**) and B (**51**)	Nitrogen-containing: cyclic peptides	Antibacterial activity	[39]
Fusarium graminearum	Marine-derived fungus	7-Hydroxy-3-(2-hydroxy-propyl)-5-methyl-epiisochromen-1-one (**117**)	Polyketides: α-pyrones	-	[39]
Fusarium graminearum	Plant pathogenic fungus	Furarin Y (**171**)	Polyketides: others	Cytotoxic activity	[34]
Fusarium graminearum	Plant pathogenic fungus	Fusaranes A (**172**) and C (**173**)	Polyketides: others	Antibacterial activity	[87,88]
Fusarium graminearum	Marine-derived fungus	Fusariellins M (**175**) and N (**176**)	Polyketides: others	Inhibitory activity on protein tyrosine phosphatase B	[90]
Fusarium graminearum	Plant pathogenic fungus	Gramiketides A (**191**) and B (**192**)	Polyketides: others	-	[94]
Fusarium graminearum	Marine-derived fungus	Kaneoheoic acids H (**199**) and I (**200**)	Polyketides: others	Antibacterial and cytotoxic activities	[39]
Fusarium graminearum	Plant pathogenic fungus	Protofusarin (**207**)	Polyketides: others	-	[96]
Fusarium graminearum	Plant pathogenic fungus	Fusarane B (**221**)	Terpenoids: sesquiterpenoids	Cytotoxic activity	[88]
Fusarium graminearum	Plant pathogenic fungus	Tricinolone (**247**); Tricinolonoic acid (**248**)	Terpenoids: sesquiterpenoids	-	[96]
Fusarium guttiforme	Plant endophytic fungus	Fusagunolics A (**274**) and B (**275**)	Phenolics	Anti-inflammatory activity	[112]
Fusarium lateritium	Plant endophytic fungus	Acuminatums E (**36**) and F (**37**)	Nitrogen-containing: cyclic peptides	Antifungal activity	[45]

Fusarium lateritium	Insect-derived fungus	Microsphaeropsisins D (**232**) and E (**233**)	Terpenoids: sesquiterpenoids	Antifungal activity	[103]
Fusarium napiforme	Mangrove-derived fungus	6-Hydroxiy-astropaquinone B (**155**); Astropaquinone D (**156**)	Polyketides: quinones	Antibacterial and phytotoxic activities	[84]
Fusarium oxysporum	Plant endophytic fungus	(*S*,*E*)-Methyl-2-(2,4-dimethylhex-2-enamido)acetate (**10**)	Nitrogen-containing: amides	-	[29]
Fusarium oxysporum	Plant endophytic fungus	Enniatin W (**45**)	Nitrogen-containing: cyclic peptides	Cytotoxic activity	[51]
Fusarium oxysporum	Plant endophytic fungus	Fusaroxazin (**96**)	Nitrogen-containing: others	Cytotoxic and antimicrobial activities	[65]
Fusarium oxysporum	Plant endophytic fungus	Fusariumin D (**129**)	Polyketides: γ-pyrones	Antibacterial activity	[77,78]
Fusarium oxysporum	Plant endophytic fungus	Neovasifuranones A (**145**) and B (**146**)	Polyketides: furanones	Antibacterial activity	[82]
Fusarium oxysporum	Soil-derived fungus	Cosmosporasides F–H (**208–210**)	Terpenoids: sesquiterpenoids	Antibacterial, cytotoxic and anti-inflammatory activities	[97]
Fusarium oxysporum	Plant endophytic fungus	Fusariumin C (**265**)	Terpenoids: others	Antibacterial activity	[78]
Fusarium oxysporum f.sp. *radicis-lycopersici*	Plant pathogenic fungus	Fusarium acids A–H (**253–260**)	Terpenoids: diterpenoids	Inhibition on hypocotyl and root elongation of tomato seedlings	[106]
Fusarium phaeoli	Plant endophytic fungus	Ergost-5,22*E*-dien-3β-oleate-20-ol (**269**)	Steroids	-	[110]
Fusarium proliferatum	Fungal stroma-derived fungus	Proliferatins A–C (**30–32**)	Nitrogen-containing: amides	Anti-inflammatory activity	[43]
Fusarium proliferatum	Plant endophytic fungus	Cyclo-(L-Trp-L-Phe-L-Phe) (**44**)	Nitrogen-containing: cyclic peptides	Cytotoxic and antibacterial activities	[50]
Fusarium proliferatum	Plant endophytic fungus	Fusarone A (**61**)	Nitrogen-containing: pyridones	Cytotoxic and antibacterial activities	[50]
Fusarium proliferatum	Plant endophytic fungus	Ethyl 3-indoleacetate (**64**)	Nitrogen-containing: indoles	Cytotoxic and antibacterial activities	[50]
Fusarium proliferatum	Plant pathogenic fungus	Proliferapyrone A (**122**)	Polyketides: α-pyrones	-	[75]
Fusarium proliferatum	Mangrove-derived fungus	Asperpentenones B (**166**) and C (**167**); Phomaligol J (**168**); Talaketides G (**169**) and H (**170**)	Polyketides: others	Cytotoxic activity	[86]
Fusarium proliferatum	Plant pathogenic fungus	Fusariumnols A (**188**) and B (**189**)	Polyketides: others	Antibacterial activity	[93]
Fusarium proliferatum	Plant pathogenic	Proliferic acids A–E (**202–206**)	Polyketides: others	Phytotoxic	[75]

	fungus			activity	
Fusarium proliferatum	Plant endophytic fungus	3β-Hydroxy-β-acorenol (**230**)	Terpenoids: sesquiterpenoids	-	[64]
Fusarium proliferatum	Soil-derived fungus	Proliferacorins A–M (**234–246**)	Terpenoids: sesquiterpenoids	-	[104]
Fusarium sacchari	Plant pathogenic fungus	Fusarochromene (**22**)	Nitrogen-containing: amides	-	[38]
Fusarium sambucinum	Plant endophytic fungus	Amoenamide C (**38**); Sclerotiamide B (**39**)	Nitrogen-containing: cyclic peptides	Antimicrobial and larvicidal activities	[46]
Fusarium solani	Marine-derived fungus	Fusarin L (**16**)	Nitrogen-containing: amides	Anti-inflamm atory activity	[33]
Fusarium solani	Mangrove-derived fungus	Fusaricates H–K (**53–56**)	Nitrogen-containing: pyridines	-	[55]
Fusarium solani	Plant endophytic fungus	Fusopoltides B (**115**) and C (**116**)	Polyketides: α-pyrones	-	[73]
Fusarium solani	Mangrove-derived fungus	Fusolanonones A (**130**) and B (**131**)	Polyketides: γ-pyrones	Antibacterial activity	[55]
Fusarium solani	Plant endophytic fungus	6-((9*R*,11*R*,*E*)-13-Hydroxy-9,1 1-dimethyloct-7-en-7-yl)-2-me thoxy-4*H*-pyran-4-one (**132**)	Polyketides: γ-pyrones	Neuroprotecti ve activity	[79]
Fusarium solani	Plant endophytic fungus	Fusaspirols A–D (**139–142**)	Polyketides: furanones	Osteoclastic differentiatio n activity	[73]
Fusarium solani	Plant endophytic fungus	Fusopoltides D (**143**) and E (**144**)	Polyketides: furanones	-	[81]
Fusarium solani	Plant endophytic fungus	Fusaridioic acid E (**174**)	Polyketides: others	Anti-inflamm atory activity	[89]
Fusarium solani	Marine-derived fungus	Fusarins G–K (**177–181**)	Polyketides: others	Anti-inflamm atory activity	[33]
Fusarium solani	Marine-derived fungus	Fusarisolins A–E (**182–186**)	Polyketides: others	Inhibition on HMG-CoA synthase gene expression	[91]
Fusarium sporotrichioides	Plant pathogenic fungus	HT2-3-*O*-α-glucoside (**228**); HT2-4-*O*-α-glucoside (**229**)	Terpenoids: sesquiterpenoids	-	[101]
Fusarium sporotrichioides	Plant endophytic fungus	8-(2-Methylbutyryl)-neosolani ol (**231**)	Terpenoids: sesquiterpenoids	-	[102]
Fusarium tricinctum	Plant endophytic fungus	Fusaritricines A–I (**78–86**)	Nitrogen-containing: imidazoles	Antibacterial activity	[62]
Fusarium tricinctum	Plant endophytic fungus	(+)-Fusaritricine J (**87**), (-)-fusaritricine J (**88**), and fusaritricines K–P (**89–94**)	Nitrogen-containing: imidazoles	Antibacterial activity	[63]
Fusarium tricinctum	Plant endophytic fungus	6-((2*S*,3*S*)-2,3-Dihydroxybutan -2-yl)-3-methyl-2*H*-pyran-2-on e (**99**); 6-((2*R*,3*R*)-2,3-Dihydroxybuta n-2-yl)-3-methyl-2*H*-pyran-2-o ne (**100**); 6-((2*S*,3*R*)-2,3-Dihydroxybuta n-2-yl)-3-methyl-2*H*-pyran-2-o ne (**101**);	Polyketides: α-pyrones	Cytotoxic activity	[66]

		6-((2*R*,3*S*)-2,3-Dihydroxybutan-2-yl)-3-methyl-2*H*-pyran-2-one (**102**)			
Fusarium tricinctum	Plant endophytic fungus	Dihydrolateropyrone (**103**)	Polyketides: α-pyrones	-	[67]
Fusarium tricinctum	Plant endophytic fungus	Fusaritricins A (**109**/**110**) and B–D (**111–113**)	Polyketides: α-pyrones	Antibacterial activity	[71]
Fusarium tricinctum	Plant endophytic fungus	Fusatricinones A–D (**161–164**)	Polyketides: quinones	-	[67]
Fusarium tricinctum	Plant endophytic fungus	(*R*,2*E*,4*E*)-6-((2*S*,5*R*)-5-Ethyltetrahydrofuran-2-yl)-6-hydroxy-4-methylhexa-2,4-dienoic acid (**212**); (*S*,2*E*,4*E*)-6-((2*S*,5*R*)-5-Ethyltetrahydrofuran-2-yl)-6-hydroxy-4-methylhexa-2,4-dienoic acid (**213**)	Terpenoids: sesquiterpenoids	Cytotoxic activity	[98]
Fusarium verticillioides	Plant endopytic fungus	Fusaisocoumarin A (**106**)	Polyketides: α-pyrones	Antifungal activity	[69]
Fusarium verticillioide	Marine fish-derived fungus	Fusaritide A (**187**)	Polyketides: others	Reduced cholesterol uptake	[92]
Fusarium sp.	Entomogenous fungus	Deacetyl fusarochromene (**2**); 4′-*O*-Acetyl fusarochromanone (**3**)	Nitrogen-containing: amines	Antimalarial activity	[25]
Fusarium sp.	Plant endophytic fungus	(3*E*,7*E*)-11,12-Dihydroxy-4,8,12-trimethyltrideca-3,7-dienamide (**9**)	Nitrogen-containing: amides	-	[28]
Fusarium sp.	Plant endophytic fungus	DihydroNG393 (**11**); Dihydrolucilactaene (**12**); 13α-Hydroxylucilactaene (**13**)	Nitrogen-containing: amides	Antimalarial activity	[30]
Fusarium sp.	Marine-derived fungus	Fusaindoterpene A (**14**)	Nitrogen-containing: amides	-	[31]
Fusarium sp.	Soil-derived fungus	Fusaramin (**15**)	Nitrogen-containing: amides	Antibacterial and antimitochondrial activity	[32]
Fusarium sp.	Plant endophytic fungus	Fusaribenzamide A (**21**)	Nitrogen-containing: amides	Antifungal activity	[37]
Fusarium sp.	Plant endophytic fungus	8(*Z*)-Lucilactaene (**24**); 4(*Z*)-Lucilactaene (**25**)	Nitrogen-containing: amides	Anti-inflammatory activity	[40]
Fusarium sp.	Marine-derived fungus	*N*-({4-[(3-methylbut-2-en-1-yl)oxy]phenyl}acetyl)glycine (**26**); Methyl *N*-({4-[(3-methylbut-2-en-1-yl)oxy]phenyl}acetyl)glycinate (**27**)	Nitrogen-containing: amides	-	[41]
Fusarium sp.	Plant endophytic fungus	Prelucilactaenes G (**28**) and H (**29**)	Nitrogen-containing: amides	Antimalarial activity	[42]
Fusarium sp.	Plant endophytic fungus	Beauvervin H (**41**)	Nitrogen-containing: cyclic peptides	Cytotoxic activity	[48]
Fusarium sp.	Plant endophytic	Beauvericins M (**42**) and N	Nitrogen-containing:	-	[49]

	fungus	(**43**)	cyclic peptides		
Fusarium sp.	Marine-derived fungus	(+)-Fusaspoid A (**71**) and (–)-Fusaspoid A (**72**)	Nitrogen-containing: indoles	-	[61]
Fusarium sp.	Marine-derived fungus	Fusaindoterpene B (**73**); Fusarindoles A–C (**74–76**); Isoalternatine A (**77**)	Nitrogen-containing: indoles	Inhibitory activity on Zika virus	[31]
Fusarium sp.	Plant endophytic fungus	Fusaristatins D–F (**47–49**)	Nitrogen-containing: cyclic peptides	-	[53]
Fusarium sp.	Entomogenous fungus	Climacomontaninate D (**52**)	Nitrogen-containing: pyridines	-	[54]
Fusarium sp.	Marine-derived fungus	Fasaripyridines A (**57**) and B (**58**)	Nitrogen-containing: pyridines	Antimicrobial and cytotoxic activities	[56]
Fusarium sp.	Marine-derived fungus	(+)-Fusaspoid A (**71**) and (–)-Fusaspoid A (**72**)	Nitrogen-containing: indoles	-	[61]
Fusarium sp.	Marine-derived fungus	Fusaindoterpene B (**73**); Fusarindoles A–C (**74–76**); Isoalternatine A (**77**)	Nitrogen-containing: indoles	Inhibitory activity on Zika virus	[31]
Fusarium sp.	Plant endophytic fungus	Secobeauvericin A (**97**)	Nitrogen-containing: others	-	[49]
Fusarium sp.	Plant endophytic fungus	(7*S*,8*R*)-Chlamydospordiol (**98**)	Polyketides: α-pyrones	-	[53]
Fusarium sp.	Plant endophytic fungus	Fupyrones A (**104**) and B (**105**)	Polyketides: α-pyrones	-	[68]
Fusarium sp.	Plant endophytic fungus	Fusaripyrones C (**107**) and D (**108**)	Polyketides: α-pyrones	-	[70]
Fusarium sp.	Intestinal fungus	Fusintespyrone A (**114**)	Polyketides: α-pyrones	Antifungal activity	[72]
Fusarium sp.	Marine-derived fungus	Karimunone A (**121**)	Polyketides: α-pyrones	Antibacterial activity	[74]
Fusarium sp.	Desert-derived fungus	Fusapyrone A (**123**)	Polyketides: γ-pyrones	Cytotoxic activity	[76]
Fusarium sp.	Marine-derived fungus	Fusaresters A–E (**124–128**)	Polyketides: γ-pyrones	Inhibitory activity on protein tyrosine phosphatase	[77]
Fusarium sp.	Plant endophytic fungus	1-Methoxylfusarnaphthoquinone A (**157**); 1-Dehydroxysolaninol (**158**); 5-Dehydroxysolaninol (**159**); Fusarnaphthoquinone D (**160**)	Polyketides: quinones	Cytotoxic activity	[85]
Fusarium sp.	Marine-derived fungus	Karimunone A (**165**)	Polyketides: quinones	-	[74]
Fusarium sp.	Entomogenous fungus	Fusariumtrin A (**190**)	Polyketides: others	-	[54]
Fusarium sp.	Marine-derived fungus	Kaneoheoic acids A–F (**193–198**)	Polyketides: others	-	[95]
Fusarium sp.	Plant endophytic fungus	Fusanoids A–G (**214–220**)	Terpenoids: sesquiterpenoids	Cytotoxic activity	[99]
Fusarium sp.	Plant endophytic fungus	Fusarchlamols A–F (**222–227**)	Terpenoids: sesquiterpenoids	Antifungal activity	[100]

Fusarium sp.	Plant endophytic fungus	3β,16α-Dihydroxy-9,15-cyclo-gibberellin A9 (**249**); 7α-Methoxy-6,7-lactone-gibberellin A12 (**250**); 7β-Methoxy-6,7-lactone-gibberellin A12 (**251**); 16α-Hydroxy-9-ene-gibberellin A14 (**252**)	Terpenoids: diterpenoids	Promoting effect on the seedling growth	[105]
Fusarium sp.	Plant endophytic fungus	Integracide L (**262**)	Terpenoids: triterpenoids	Lipoxygenase inhibitory activity	[108]
Fusarium sp.	Plant endophytic fungus	(*E*)-9,10-Dihydroxy-2,6,10-trimethylundec-5-enoic acid (**263**); (*E*)-8,9-Dihydroxy-1-methoxy-5,9-dimethyldec-4-en-2-one (**264**)	Terpenoids: others	-	[28]
Fusarium sp.	Mouse intestinal fungus	Cerevisterolside A (**266**)	Steroids	Antifungal activity	[72]
Fusarium sp.	Plant endophytic fungus	Fusaristerols B–D (**270–272**)	Steroids	Anti-inflammatory activity	[111]
Fusarium sp.	Plant endophytic fungus	4-Hydroxy-4-methylpentyl 2-(4-hydroxyphenyl) acetate (**276**)	Phenolics	Phytotoxic activity	[113]
